# Gain of function mutant p53 proteins cooperate with E2F4 to transcriptionally downregulate RAD17 and BRCA1 gene expression

**DOI:** 10.18632/oncotarget.2587

**Published:** 2015-02-05

**Authors:** Fabio Valenti, Federica Ganci, Giulia Fontemaggi, Andrea Sacconi, Sabrina Strano, Giovanni Blandino, Silvia Di Agostino

**Affiliations:** ^1^ Translational Oncogenomic Unit, Molecular Medicine Area, Regina Elena National Cancer Institute, Rome 00144, Italy; ^2^ Molecular Chemoprevention Group, Molecular Medicine Area, Regina Elena National Cancer Institute, Rome 00144, Italy

**Keywords:** mutant p53, gain-of-function, genomic instability, DNA damage response, BRCA1, RAD17

## Abstract

Genomic instability (IN) is a common feature of many human cancers. The *TP53* tumour suppressor gene is mutated in approximately half of human cancers. Here, we show that BRCA1 and RAD17 genes, whose derived proteins play a pivotal role in DNA damage repair, are transcriptional targets of gain-of-function mutant p53 proteins. Indeed, high levels of mutp53 protein facilitate DNA damage accumulation and severely impair BRCA1 and RAD17 expression in proliferating cancer cells. The recruitment of mutp53/E2F4 complex onto specific regions of BRCA1 and RAD17 promoters leads to the inhibition of their expression. BRCA1 and RAD17 mRNA expression is reduced in HNSCC patients carrying *TP53* mutations when compared to those bearing wt-p53 gene. Furthermore, the analysis of gene expression databases for breast cancer patients reveals that low expression of DNA repair genes correlates significantly with reduced relapse free survival of patients carrying TP53 gene mutations. Collectively, these findings highlight the direct involvement of transcriptionally active gain of function mutant p53 proteins in genomic instability through the impairment of DNA repair mechanisms.

## INTRODUCTION

Upon DNA insults, stabilization of the tumour suppressor p53 leads to transcription of genes involved in cell cycle arrest, senescence, DNA repair and apoptosis to prevent accumulation of unrepaired DNA and propagation of mutated DNA [1-3]. *TP53* gene is mutated in more than half of all human cancers [4]. P53 mutations disrupt wt-p53 tumour suppressive functions and also confer new oncogenic properties (GOF) that contribute to growth advantage of tumour cells [2, 3]. Many evidences pointed out that GOF mutp53 proteins promote invasion, metastasis and structural chromosomal changes resulting in high levels of genomic instability (IN) in different tumours [5-8]. Concerning the molecular mechanisms through which mutp53 proteins exert their oncogenic functions, we and others previously characterized their ability to modulate gene expression through interaction with other transcription factors, such as NF-Y, E2F1, NF-kB, ZEB1, SP1, ETS1 and VDR [3, 9-15]. Mutp53 proteins also bind to p53 family members, p63 and p73 impairing their transcriptional activity and consequently their anti-tumoural effects [16-19]. We documented the existence of an oncogenic autoregulatory feedback loop that includes the Polo-like kinase2 (*Snk/Plk2*), a regulator of centrosomal checkpoint, and mutp53 proteins where Plk2 binds to and phosphorylates mutp53, thereby potentiating its oncogenic activities [20].

The homologous recombination (HR) and non-homologous end joining (NHEJ) are the DNA repair mechanisms present in the cells [21]. Mutp53 was shown to bind to and inhibit the DNA repair binding protein MRE11 limiting the phosphorylation and activation of Ataxia Telangiectasia Mutated protein (ATM) [22]. This event resulted in bypassing the G2/M DNA damage checkpoint causing a reduced maintenance of the genetic information [22].

BRCA1 is a nuclear tumour suppressor phosphoprotein that plays a role in maintaining genomic stability. It is involved in gene transcription, cell cycle arrest and DNA damage repair [23, 24]. Cells lacking BRCA1 expression showed defects in DNA repair by homologous recombination [24]. BRCA1 mutation accounts for nearly half of familial breast cancers, but BRCA1 is also down-regulated in sporadic breast tumors without germline mutation [25]. *hRAD17* is the homologue of the *Rad17* gene of *S. pombe.* RAD17 protein is required for cell cycle arrest and DNA damage repair in response to DNA damaging insults [1, 23]. In response to DNA damage, RAD17 recruits the Rad9-Hus1-Rad1 (9-1-1) complex, probably by acting as a clamp loader to load the 9-1-1 complex onto DNA damage sites [1]. Both BRCA1 and RAD17 proteins are key signal transducers during checkpoint activation in the response to DNA DSBs [1, 26]. *BRCA1* and *RAD17* mutations are rarely detected in sporadic tumours. While the reduction of B*RCA1* and *RAD17* expression in sporadic cancers is well established, the molecular mechanisms by which their expression is downregulated in tumour cells are still unclear [27-29].

Here, we show that transcriptional activity of GOF mutp53 proteins plays a role in the inefficient DNA repair and consequent DNA damage accumulation in proliferating tumour cells. We found that *BRCA1* and *RAD17* genes are transcriptional targets of mutp53 proteins. Mutp53 and E2F4 proteins formed a transcriptional repressive complex that assembled onto the regulatory regions of *BRCA1* and *RAD17* genes inhibiting their expression. Moreover, BRCA1 and RAD17 transcripts are reduced specifically in *TP53* mutation-carrying tumors from head and neck squamous cell carcinoma (HNSCC) patients. HNSCC is characterized by a high grade of genomic instability and a *TP53* mutations incidence of nearly 62% [31]. Altogether, these findings highlight yet another unexplored transcriptional activity of mutp53 in DNA damage response that might hold therapeutic potential.

## RESULTS

### Mutant p53 promotes accumulation of DNA mutations in growing cells

GOF mutp53 proteins were previously implicated in promoting IN [32, 33]. Notably, ectopic expression of mutp53R172H (corresponding to human R175H) in p53-null primary mouse mammary epithelial cells and developing mouse mammary tumours resulted in aberrant centrosome amplification, multipolar mitoses and increased numbers of chromosomes [5, 7, 8, 34]. However, the molecular mechanisms underlying this oncogenic effect are not yet fully characterized. This prompted us to investigate whether the expression of mutp53 induced DNA alterations during the proliferation of tumor cells.

**Figure 1:**
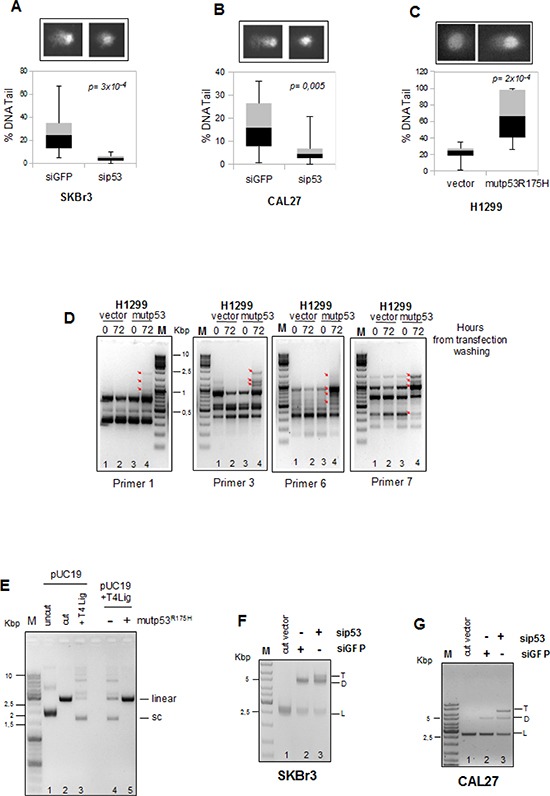
Mutant p53 promotes accumulation of DNA mutations during cell growth **(A** and **B)** SKBr3 and CAL27 cells were transiently transfected with sip53 and siGFP oligos as control and then were examined in an *in vivo* comet assay using the alkali method that detects DNA single-strand breaks, double-strand breaks and alkali-labile lesions. **(C)** H1299 cell line transfected with pcDNA3-p53R175H vector and empty pcDNA3 vector as control were analyzed for amount of DNA damaged by comet assay as described in a and b. It was used a software tool to provide an automated analysis of comet assay images (OpenCOMET; http://www.opencomet.org) [38]. The extent of DNA damage is related to the amount of DNA in the tail. The percentage of DNA in the tail is plotted in the lower panels of each figures. About 100 cells were evaluated for each sample. In all box plots, the bottom (black) and top (grey) of the box are always the first (25th percentile) and third quartiles (75h percentile), and the band inside the box is always the second quartile (the median). P-values were calculated with two-tailed t-test. Statistically significant results were with *p*-value < 0.05.**(D)** H1299 cells were transiently transfected with pCDNA3-vector and pCDNA3-mutp53R175H for 16h (over night) and then plated (time 0h) and grown for 72h. Genomic DNA purified was used as a template in the RAPD-PCR analysis to assess the presence of DNA mutations. The arrows indicate the variations in the aplified bands. M is the DNA ladder. PCR reactions were performed with the indicated primers. **(E)** Comparison of ligation products of 5′-cohesive-ended linear DNA (lane 2) in the presence of T4 DNA ligase alone (lane 3) or following pre-incubation with whole protein extracts of H1299 cells transfected with mutp53R175H or control expressing vectors (lanes 5 and 4, respectively). Numbers indicate the lanes. **(F, G)** Comparison of DNA repair products after 16h at 17°C of cellular extracts derived from SKBr3 (f) and CAL27 (g) cells transiently transfected with siGFP (lane 2) as control and sip53 (lane 3) oligos. Lane 1 represents the reaction buffer mixture with HindIII-cut vector. DNA product bands are indicated as follows: L, linear DNA; D, dimer; T, trimer. Numbers indicate the lanes.

To this end, SKBr3 breast cancer cells (endogenously expressing mutp53R175H) and CAL27 head and neck cancer cells (endogenously expressing mutp53A193T) were transfected for 18 hours with siRNAs directed to mutp53 (sip53), or control siRNAs (siGFP). After the transfection washing the cells were allowed to grow for 48 hours. *In vivo* comet assay analyses performed in these cells revealed that the mutp53 knocking-down reduced the amount of DNA damage, visualized as percentage of DNA in the tail of the comets (Figures 1A and B and Supplementary Figures 1A and B for interference control) compared to control cells [38]. To further corroborate the results of the comet assays, we evaluated histone H2AX^Ser139^ phosphorylation state, as readout of the DNA damage response [21]. As shown in Supplementary Figures 1A and B, mutp53-depleted SKBr3 and CAL27 cells (sip53) showed decreased H2AX phosphorylation rate, compared to control cells (siGFP). This indicates that mutant p53 expression correlates with increased DNA damage. Similar results were obtained in MDA-MB-468 breast cancer cells carrying endogenous mutp53R273H (Supplementary Figures 1D and E).

Furthermore, comet assay performed in H1299 cells transfected with the mutp53R175H protein expression vector (Supplementary Figure 1C) showed a significant increase in the comet formation compared to control cells (Figure 1C) with the concomitant phosphorylation of H2AX as sign of an increased DNA damage (Supplementary Figure 1C).

To study the involvement of mutp53 in these DNA alterations during cell growth we used the RAPD assay (Random Amplified Polymorphism DNA) (see Supplementary Info 1 for technical details) [35]. To this end, genomic DNA was extracted from p53-null H1299 lung adenocarcinoma cells transfected with mutp53R175H vector and empty pcDNA3 as control, collected at 0h and 72h of proliferation and analyzed by a control PCR for integrity and amount (Supplementary Figure 1F and G). The use of arbitrary, short primers in RAPD assay allowed us to observe a series of alterations occurring in the genome of H1299 cells during proliferation, as revealed by changes in the PCR amplification patterns of the various primer sets (Figure 1D, red arrows).

We observed very small changes in the PCR amplification pattern of control cells (vector) during the time (Figure 1D, lanes 2 vs 1 in each panel), while mutp53 overexpressing cells showed very marked changes with all the used primer sets (Figure 1D, lanes 4 vs 3 in each panel). This revealed the generation of inefficiently repaired DNA damage in the presence of mutant p53 in proliferating cancer cells. We reasoned that this could rely on the inability of the DNA repair machinery to work efficiently in cells carrying mutant p53 proteins.

To assess whether mutp53 could interfere with the DNA repair machinery, we investigated *in vitro* the involvement of mutp53 in DNA double-strand breaks (DSBs) repair. Cell-free extracts were shown to be an adequate experimental system for the study of DNA repair activity using the *T4 DNA ligase assay* [36, 37]. pUC19 vector was linearized with *HindIII* to generate 5′-cohesive ends (Figure 1E, lane 2) and used as substrate. Incubation of 5′-cohesive-ended linear DNA (2, 6 Kb) with the cellular extracts derived from H1299 cells transfected with mutp53R175H protein, in the presence of T4 ligase, resulted in the inhibition of the production of the supercoiled and high oligomeric forms, compared to the H1299 cells transfected with the empty vector (Figure 1E, lanes 4–5 and Supplementary Figure 2A for quantification).

To ensure that the above-described inhibitory effect on linearized plasmid ligation was not aspecific, we overexpressed wild-type p53 or mutp53R175H proteins in p53-null H1299 cells and used the derived cell lysates for the *in vitro* ligation assay (Supplementary Figure 2B). As shown in Supplementary Figure 2C we observed that only the lysate of mutp53R175H-expressing H1299 (lane 6), and not that of wt-p53-expressing cells (lane 5), showed an inhibitory effect on ligation.

To exclude that mutp53R175H has a direct inhibitory effect on the T4 ligase enzymatic activity, we performed *in vitro* ligation assays using GST-purified proteins. For this purpose we purified GST-mutp53R175H protein and GST alone, as negative control (Supplementary Figure 2D). As shown in Supplementary Figure 2E, no direct effect on T4 ligase enzymatic activity was observed in presence of increasing amounts of purified GST-mutp53R175H protein (100, 200 and 500 ng; lanes 7, 8 and 9, respectively) or GST alone (lanes 4, 5 and 6).

To study the effect of endogenous mutp53 on the cellular DNA repair activity, pUC19 linearized was incubated with total cell extracts derived from breast and head & neck cancer cell lines (SKBr3 and CAL27, respectively) whose endogenous mutp53 expression was selectively knocked-down (Supplementary Figures 3A and B). As shown in Figure 1F and G, mutp53-depleted extracts (sip53, lane 3) showed higher efficiency in DNA repair activity than control extracts (siGFP, lane 2), as proved by the increased formation of di-oligomers (D) and tri-oligomers (T) and the decreased linearized form (L). These findings indicated that expression of mutp53 protein results in inhibition of DNA repair activity.

Overall, these findings document that mutp53 activity favors accumulation of DNA mutations by interfering with DNA repair machinery.

### Depletion of mutant p53 affects BRCA1 and RAD17 gene expression

Accumulated genomic mutations from improperly repaired DNA, particularly at the sites of caretaker genes, might result in malignant transformation [32, 39, 40]. Notably, it was reported that cultured tumour cells exhibit a basal level of DNA damage in their proliferation [23, 41, 42].

We above showed that tumour cells carrying mutp53 proteins accumulated DNA mutations (Figure 1D) and the ability to repair DSBs damage was re-gained in the absence of mutp53 expression (Figures 1E-G). To further investigate the role of mutp53 in promoting IN we studied whether mutp53 proteins could control the expression of genes involved in the DNA repair mechanism. We considered the expression of BRCA1 and RAD17, as they are key signal transducers, and CHK1 protein as kinase effector during checkpoint activation in the response to DNA DSBs [1, 26]. All *BRCA1*, *RAD17* and *Chk1* mutations are rarely detected in sporadic tumours [26].

**Figure 2:**
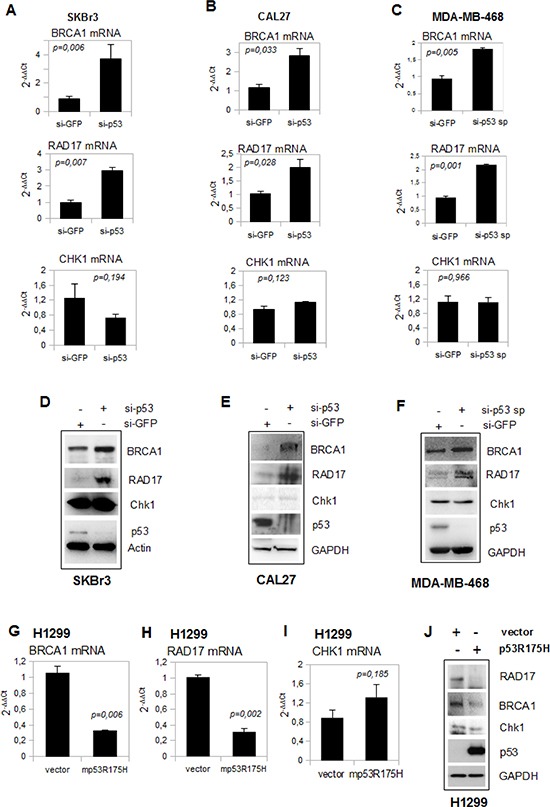
Depletion of mutant p53 affects BRCA1 and RAD17 gene expression **(A-C)** Quantitative RT-PCR (qRT-PCR) analysis of *BRCA1, RAD17* and *CHK1* expression in SKBr3 (a), CAL27 (b) and MDA-MB-468 (c) cell lines transiently knocked-down for p53 expression with the indicated siRNA oligonucleotides. Standard deviation in each experiment was derived from the analysis of biological triplicates. **(D-F)** Western blot analysis was performed with 50 μg of whole cell extracts and probed with the indicated antibodies from si-p53 SKBr3 (d), sip53 CAL27 (e) and si-p53 sp MDA-MB-468 (f) and from si-GFP cells as control. Cells were grown for 48 h after transfection. **(G-I)** qRT-PCR analysis for *BRCA1*(g)*, RAD17* (h) and *CHK1* (i) expression was carried out from H1299 cells transiently transfected for 48 h with 2 μg of pCDNA3-mutp53R175H. Western blotting analysis **(J)** from these cells was performed with 50 μg of whole cell extracts and probed with the indicated antibodies. The experiments were produced in biological triplicates. P-values of the shown qRT-PCR experiments were calculated with two-tailed t-test. Statistically significant results were with *p*-value < 0.05.

The depletion of mutp53 protein by the transfection with the indicated siRNAs strongly increased the expression of BRCA1 and RAD17 transcripts in three cell lines (SKBr3, CAL27 and MDA-MB-468). Unlike BRCA1 and RAD17, the expression of Chk1 mRNA was not modulated (Figures 2A-C). The expression of BRCA1, RAD17 and Chk1 proteins, analyzed by western blotting, correlated with that of the transcripts (Figures 2D-F).

Moreover, ectopic expression of mutp53R175H in H1299 cells leads to the reduction of BRCA1 and RAD17 transcripts (Figures 2G and H) and protein (Figure 2J) levels, while Chk1 mRNA and protein expression remained unchanged (Figures 2I and J). A reduction of BRCA1 and RAD17 expression was also observed in H1299 cells ectopically expressing mutp53R273H and mutp53D281G proteins (Supplementary Figures 4A-C).

Depletion of wt-p53 expression in MCF7 breast cancer cell line did not affect the expression of RAD17 and BRCA1 transcripts (Supplementary Figures 4D-F).

### Mutant p53 protein binds to BRCA1 and RAD17 gene promoters

To investigate how mutp53 affects BRCA1 and RAD17 expression we evaluated its potential recruitment on their regulatory regions.

**Figure 3:**
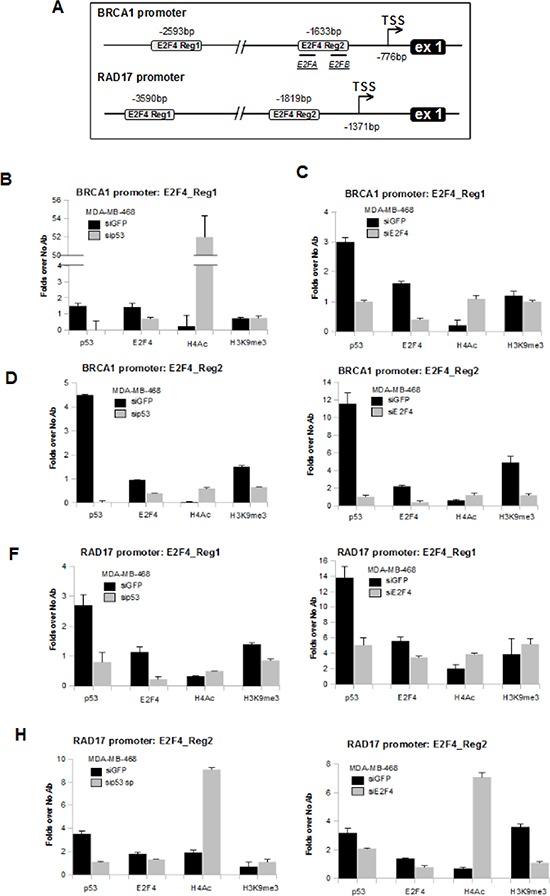
Mutant p53 protein binds to BRCA1 and RAD17 gene promoters **(A)** Schematic representation of BRCA1 and RAD17 promoter gene regions containing E2F4 consensus box sequences analyzed in ChIP assays. BRCA1 promoter: two regions at 2593 bp and 1633 bp upstream the first exon of BRCA1 gene. *E2FA* and *E2FB* is the region previously characterized by the Glazer's group [43]. RAD17 promoter: two regions at 3590 bp and 1819 bp upstream the first exon of RAD17 gene. The TSS is indicated for each gene by Eponine software prediction [63]. **(B-I)** Cross-linked chromatin derived from si-p53-MDA-MB-468 (b, d, f, h) and from siE2F4-MDA-MB-468 (c, e, g, i) cells with their respective siGFP-transfected control, was immunoprecipitated with the indicated antibodies or in the absence of antibody, and analyzed by qRT-PCR with specific primers for the indicated regions in the Figure 3A. Each point of the experiment was carried out in biological duplicate and each reaction of each sample in qRT-PCR was carried out in technical replicate. The P-values were calculated with two-tailed t-test. Statistically significant results were with *p*-value < 0.05.

We and others have shown that mutp53 binds to the promoters of its target genes through the cooperation with other transcription factors, such as E2F1, NF-Y, Sp1 and others [9-15]. Since mutant p53 down-regulates BRCA1 and RAD17 expression we analyzed their promoters looking for consensus of transcriptional repressors. It was previously reported that E2F4 functions as a basal repressor of *BRCA1* expression and occupies specific DNA consensus sequences onto BRCA1 promoter [43]. E2F4 is a component of the repressive DREAM complex, wich includes other co-factors [44]. By using MatInspector software (http://www.genomatix.de) we identified E2F4 consensus sequences on both BRCA1 and RAD17 promoters (Figure 3A).

The E2F4 Region 2 of BRCA1 promoter shown in Figure 3A coincides with the consensus sequences previously characterized by the Glazer's group (indicated as *E2FA* and *E2FB* in the Figure 3A; Supplemental File 1) [43].

Chromatin immunoprecipitation analyses (ChIP) of si-GFP, si-p53 and si-E2F4 MDA-MB-468 cells (Supplementary Figures 5A and B) revealed that E2F4 and mutp53 proteins were recruited onto E2F4 consensus sites of Region 1 of BRCA1 promoter (−2593 bp; Figures 3B and C), Region 2 of BRCA1 promoter (−1633 bp; Figures 3D and E), Region 1 of RAD17 promoter (−3590 bp; Figures 3F and G) and Region 2 of RAD17 promoter (−1819 bp; Figures 3H and I). These recruitments correlated with trimethylation of histone H3 in lysine 9 on both BRCA1 and RAD17 promoters (Figures 3B-I), in agreement with the observed transcriptional repression of BRCA1 and RAD17 promoters.

Notably, the recruitment mutp53 was generally impaired in si-p53, as expected, and in si-E2F4 MDA-MB-468 cells on both BRCA1 and RAD17 promoters (Figures 3B-I). Also E2F4 recruitment on these promoters was decreased after mutp53 depletion (Figures 3B, D, F and H) as well as after depletion of E2F4 itself, as expected (Figures 3C, E, G and I). Furthermore, we assessed an increased histone H4 acetylation and a reduced histone H3K9 trimethylation rates (Figures 3B-I) on the majority of the analyzed genomic regions following interference of mutp53 or E2F4. This paired with the observed transcriptional induction of BRCA1 and RAD17 in these conditions (Figures 2A-C).

Transactivation assays showed that depletion of endogenous mutp53 in SKBr3 cells caused the increase of the luciferase activity of a reporter vector enclosing the BRCA1 Reg2 promoter (Supplementary Figures 5C and E). Similar results were obtained upon E2F4 depletion (Supplementary Figures 5C and E). No induction of LUC activity was observed in si-p53 and si-E2F4-SKBr3 cells when a construct with mutated Reg2 of BRCA1 promoter was used (Supplementary Figure 5D).

Altogether these findings show that gain of function mutp53 protein transcriptionally represses the expression of BRCA1 and RAD17 genes through the cooperation with the transcription factor E2F4.

### Mutant p53 and E2F4 proteins bind concomitantly BRCA1 and RAD17 promoters

**Figure 4:**
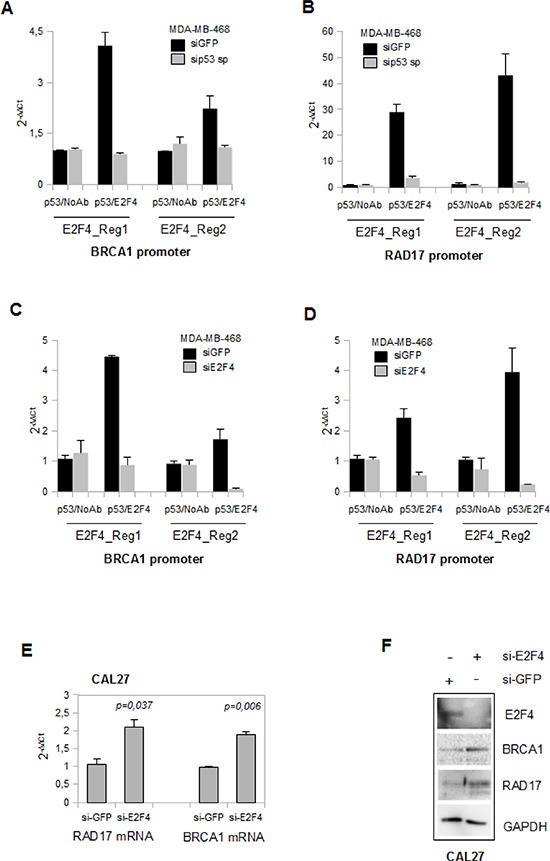
Mutant p53 and E2F4 proteins bind concomitantly BRCA1 and RAD 17 promoters **(A-D)** Re-ChIP assays of si-p53-MDA-MB-468 (a and b) and of si-E2F4-MDA-MB-468 (c and d) cells with their siGFP-transfected control, using the indicated antibodies. The analysis performed by qPCR was employed using specific primers for the previously indicated regions in the Figure 3A. **(E)** qRT-PCR analysis of *RAD17* and *BRCA1* expression of transiently transfected CAL27 cells (for 48 h with 150pmol of si-GFP and si-E2F4 oligogonucleotides) were done as biological triplicates. P-values were calculated with two tailed t-test. Statistically significant results were with *p*-value < 0.05. **(F)** The western blot analysis of 40 μg derived from protein lysates of CAL27 cells previously used in (e) was performed to evaluate the expression of the indicated proteins.

As both mutp53 and E2F4 proteins bind E2F4 consensus sequences onto RAD17 and BRCA1 promoters, we aimed to investigate whether their binding was simultaneous and represented the formation of a novel repressive competent complex. To this end we performed sequential ChIP (Re-ChIP) analyses in MDA-MB-468 cells (Figures 4A-D). We found that mutp53 and E2F4 were simultaneously present on Regions 1 and 2 of both BRCA1 (Figures 4A and C) and RAD17 (Figures 4B and D) promoters. As expected, depletion of mutp53 or E2F4 compromised the sequential immunoprecipitations of BRCA1 and RAD17 genomic regions (Figures 4A-D).

According to the above findings, si-RNA-mediated knocking down of E2F4 led to the resumption of RAD17 and BRCA1 mRNA transcription and protein expression (Figures 4E and F) in CAL27 cells.

### Mutant p53 and E2F4 proteins form a protein complex in tumour cells

**Figure 5:**
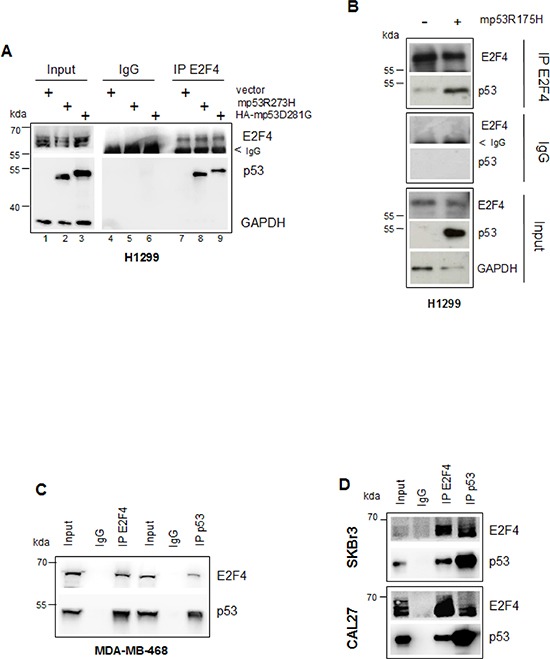
Mutant p53 and E2F4 proteins form a protein complex in tumour cells **(A, B)** H1299 cells were transfected with 2 μg of pcDNA3-p53R273H, pcDNA3HA-p53D281G (a) and pcDNA3-p53R175H (b) vectors and empty pcDNA3 as control. Immunoprecipitation of the whole cell extracts derived from these samples were performed with E2F4 antibody and preimmune rabbit serum. Cell extracts (30 μg) and immunoprecipitated samples (800 μg) were subjected to western blotting with the indicated antibodies. **(C, D)** Whole cell extracts (40 μg) and immunoprecipitations (800 μg) from MDA-MB-468 (c), SKBr3 and CAL27 cells respectively (d), immunoprecipitated with E2F4 and p53 antibodies were subjected to western blot analysis probed with the indicated antibodies.

In line with the results obtained by studying the mutp53/E2F4 occupancy onto the RAD17 and BRCA1 promoters, we assessed the existence of a floating mutp53/E2F4 protein complex. Ectopically expressed mutp53R273H, HA-mutp53D281G and mutp53R175H proteins co-precipitated with endogenous E2F4 protein (Figures 5A and B). Co-precipitation experiments performed in MDA-MB-468, SKBr3 and CAL27 cell lines documented the presence of an endogenous mutp53/E2F4 complex (Figures 5C and D).

The existence of this floating protein complex in mutp53 cancer cells, accordingly with the results of the ChIP analysis (Figures 3 and 4) suggests a direct role of transcriptionally active gain of function mutant p53 proteins in the constitutive active DNA damage signalling occurring in proliferating tumour cells.

### BRCA1 expression counteracts mutant p53 GOF activity on DNA repair assay

We aimed to investigate whether the reintroduction of BRCA1 expression in mutant p53 expressing cells recovers the ability to efficiently repair DNA.

**Figure 6:**
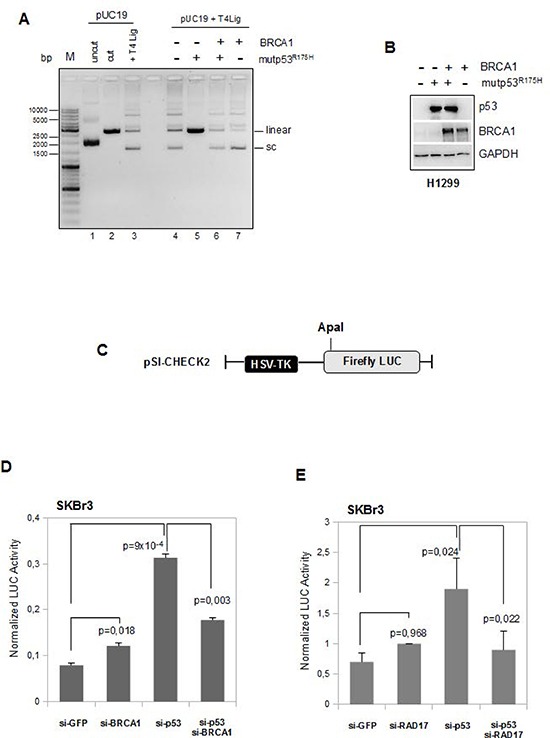
BRCA1 expression counteracts mutant p53 GOF activity on DNA repair assay **(A)** Comparison of ligation products of 5′-cohesive-ended linear DNA in the presence of T4 DNA ligase alone (lane 3) or following pre-incubation with whole protein extracts derived from H1299 cells transfected with mutp53R175H and BRCA1 expressing vectors in separate reactions (lanes 5 and 7, respectively) or in co-trasfection conditions (lane 6). **(B)** Whole protein extracts (40 μg) used in the T4 DNA ligase assay previously described were subjected to Western blot analysis and probed with the indicated antibodies. **(C-E)** SKBr3 cells were transiently transfected with ApaI-linearized pSI-CHECK2 vector (c) and with either siRNA oligos indicated in the figures (d) and (e). After 48 h from the transfection the cells were harvested and the functional changes in NHEJ were assessed measuring the Firefly Luciferase activity. Luciferase activity was expressed as (Firefly/protein amount) × (1/Renilla). *Columns*, means from two independent assays each of them was done in triplicate; *bars*, SD. *P*-values were calculated with two tailed t-test. Statistically significant results were with *p*-value < 0.05.

For this purpose, we used the *in vitro T4 DNA ligase assay* previously described [37]. As shown in Figure 6A and B, the concomitant expression of BRCA1 and mutp53R175H proteins in H1299 cells reverted the above described (Figure 1E) inhibitory effect of mutp53 on the ligation of 5′-ended linear DNA (Figure 6, lane 6 vs lane 5). Quantification of supercoiled (SC), linear, and multimeric forms generated in this assay is shown in Supplementary Figure 5F.

Moreover, co-expression of BRCA1 and mutp53R175H proteins resulted in reduced phospo-H2AX levels, compared to expression of mutp53 alone in H1299 (Supplementary Figure 5G, lane 3 and 4). This indicates that BRCA1 reintroduction was able to rescue DNA repair activity.

The different components present in some replicates of this assay were reported with their standard deviations (Supplementary Figure 5F).

An additional approach, based on the quantitative evaluation of luciferase activity following ligation of a *Firefly luciferase*-carrying vector (previously reported by [43]), was used to assess the impact of BRCA1 and RAD17 expression on the mutp53-dependent inhibition of DNA repair.

In this DNA repair assay, the pSI-CHECK2 plasmid, linearized by *ApaI* cutting in its *Firefly luciferase* gene (Figure 6C), was co-transfected along with siRNAs for mutp53, or BRCA1, or both mutp53 and BRCA1, or GFP (as control), in SKBr3 cells. Endogenous end-joining activity, resulting in the reconstitution of the luciferase gene, was detected as luciferase activity (Figure 6C and D; Supplementary Figure 5H).

We observed that mutp53 knock-down rescued the luciferase activity (si-p53 vs si-GFP, Figure 6D), while the concomitant sip53/siBRCA1 impaired this rescue (si-p53/si-BRCA1 vs si-p53, Figure 6D). The repair activity remained inhibited in siGFP- and siBRCA1-SKBr3 cells (Figures 6D). We obtained comparable results in SKBr3 cells transfected with siRNA against RAD17 mRNA (Figure 6E; Supplementary Figure 6I).

### Reduced expression of DNA repair genes correlates with mutant p53 expression in HNSCC patients

To evaluate whether the expression levels of BRCA1 and RAD17 were different by the presence or absence of *TP53* mutations in human cancer samples we carried out RT-qPCR analysis in matched tumour and normal tissues from 63 head and neck squamous cell carcinoma (HNSCC) patients, of which 32 presented tumors with a mutated *TP53* and 31 with a wild-type *TP53* gene (Supplementary Table 3). These samples belong to the cohort previously described by Ganci and colleagues, where *TP53* status was assessed by direct sequencing of exons 2 through 11 [45, 46]. In HNSCCs *TP53* mutation is a very frequent event and in our series its incidence is nearly 58%.

**Figure 7:**
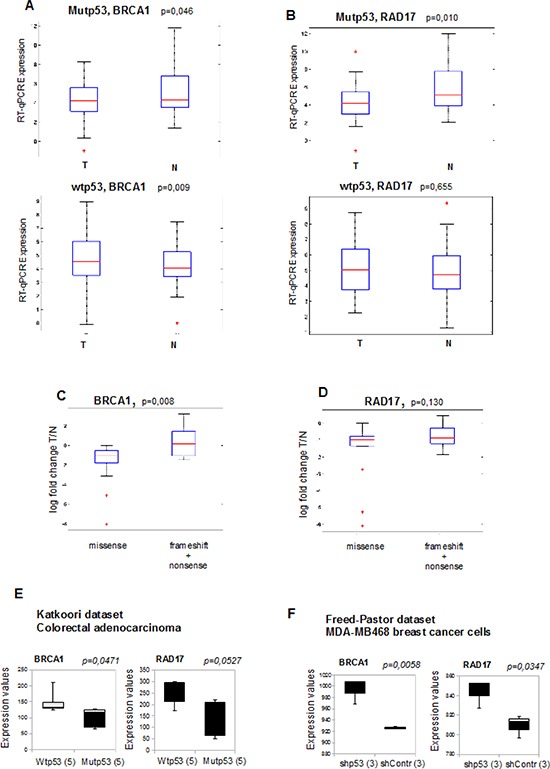
Reduced expression of DNA repair genes correlates with mutant p53 expression in cancer patients **(A, B)** BRCA1 and RAD17 mRNAs were analyzed by RT-qPCR on a group of 63 HNSCC tumor samples and on their normal counterparts. The expression of BRCA1 (a) and RAD17 (b) mRNAs (log base 2 scale) were analyzed in association of *TP53* gene mutational status in HNSCC samples. Mutp53: tumors carrying mutant p53 (n = 32); wtp53: tumors with wild-type TP53 (n = 31). **(C, D)** Association of BRCA1 (c) and RAD17 (d) mRNA expression (log fold change T/N) with tumors carrying TP53 missense mutations (leading to protein stabilization) with a selected group of tumors carrying nonsense and frameshift mutations not leading to protein stabilization (by sequence prediction analysis and by immunohistochemistry, as previously described in Ganci et al., Omics 2011). On each box, the central red mark is the median, the blue edges of the box are the 25th and 75th percentiles, the black lines extend to the most extreme data points not considered outliers, and outliers are plotted individually as +. **(E)** The box plot represents BRCA1 (left panel) and RAD17 (right panel) mRNA expression of 5 wild type p53-and 5 mutant p53-carrying colorectal adenocarcinoma patients. Data from Katkoori et al. (2012) were obtained from www.oncomine.org website.^45,46^ Data are presented as log base 2 scale. **(F)** The box plots represent BRCA1 (left panel) and RAD17 (right panel) and mRNA expression in three separate cell clones of sh-p53 (shp53) and sh-control (shContr) MDA-MB-468 breast cancer cells (mutp53R273H). Data from Freed-Pastor et al. (2012) were obtained from www.oncomine.org website [45, 47]. Data are presented as log base 2 scale. In all box plots, the bottom (black) and top (grey) of the box are always the first (25th percentile) and third quartiles (75h percentile), and the band inside the box is always the second quartile (the median).

We observed that *BRCA1* and *RAD17* were expressed at lower level in tumor samples, compared to normal counterparts, specifically in the group of patients with mutp53 (Figure 7A and B, upper box plots). The downregulation of BRCA1 and RAD17 in the mutp53 tumor samples was independent from other clinicopathological parameters in this group of patients (Supplemental Table 4).

Patients with wt-p53 tumors didn't show any significant difference for RAD17 expression between T and N (Figure 7B, lower graph), while, interestingly, BRCA1 was upregulated in wt-p53 tumors (Figure 7A, lower graph).

We also compared tumors carrying missense mutations of TP53 (usually leading to protein stabilization and gain of function) with a selected group of tumors, characterized by nonsense (NS) mutations and frameshift (FS) mutations. As shown in Figure 7C, significant lower expression levels were observed for BRCA1 in the group of tumors with missense mutations compared to the group with FS/NS mutations, and a similar trend was observed for RAD17 (Figure 7D). This result supports the hypothesis of an active repression of BRCA1 and RAD17 by mutant p53 proteins.

To further investigate this inverse correlation between the expression of mutp53 and BRCA1 and RAD17, we queried public gene expression data repositories (http://www.oncomine.org/) [47]. Analysis of the data set from Katkoori and colleagues [48] revealed that BRCA1 and RAD17 transcripts are substantially downregulated in colorectal adenocarcinoma samples carrying mutp53, compared to those with wt-p53 (Figure 7E). This finding was corroborated by the additional analysis of the data set from Freed-Pastor et al. [49] where three separate biological clones of MDA-MB-468 breast cancer cells (carrying mutp53R273H) showed lower levels of BRCA1 and RAD17 transcripts compared to the relative stable mutp53 knocked-down clones (Figure 7F).

Finally, we assessed the potential correlation between *RAD17* and *BRCA1* DNA repair genes expression and clinical outcome in a cohort of sporadic basal-like breast carcinomas (BLCs) (Supplementary Figures 6A-D). BLCs are characterized by high frequency of *TP53* mutation (92%) and high rate of genomic instability [49–52]. We analysed gene expression datasets and survival information of 478 BLC patients downloaded from GEO (http://www.ncbi.nlm.nih.gov/geo/) (Web address: www.kmplot.com) [53]. To analyse the prognostic value of DNA repair genes, patients with high and low gene expression levels were divided into two groups. The two groups were then compared in terms of relapse free survival (RFS). We found that BLC patients expressing low levels of *RAD17* and *BRCA1* genes exhibited a significant reduced relapse-free survival when compared to those expressing high levels (Supplementary Figures 6A and B). Interestingly, BLCs patients expressing high levels of *cyclin B1* and *id4* genes, that we have previously shown to be transcriptional targets of GOF mutp53 proteins [9, 11] exhibited poorer RFS compared to those with low expression (Supplementary Figures 6C and D).

## DISCUSSION

**Figure 8:**
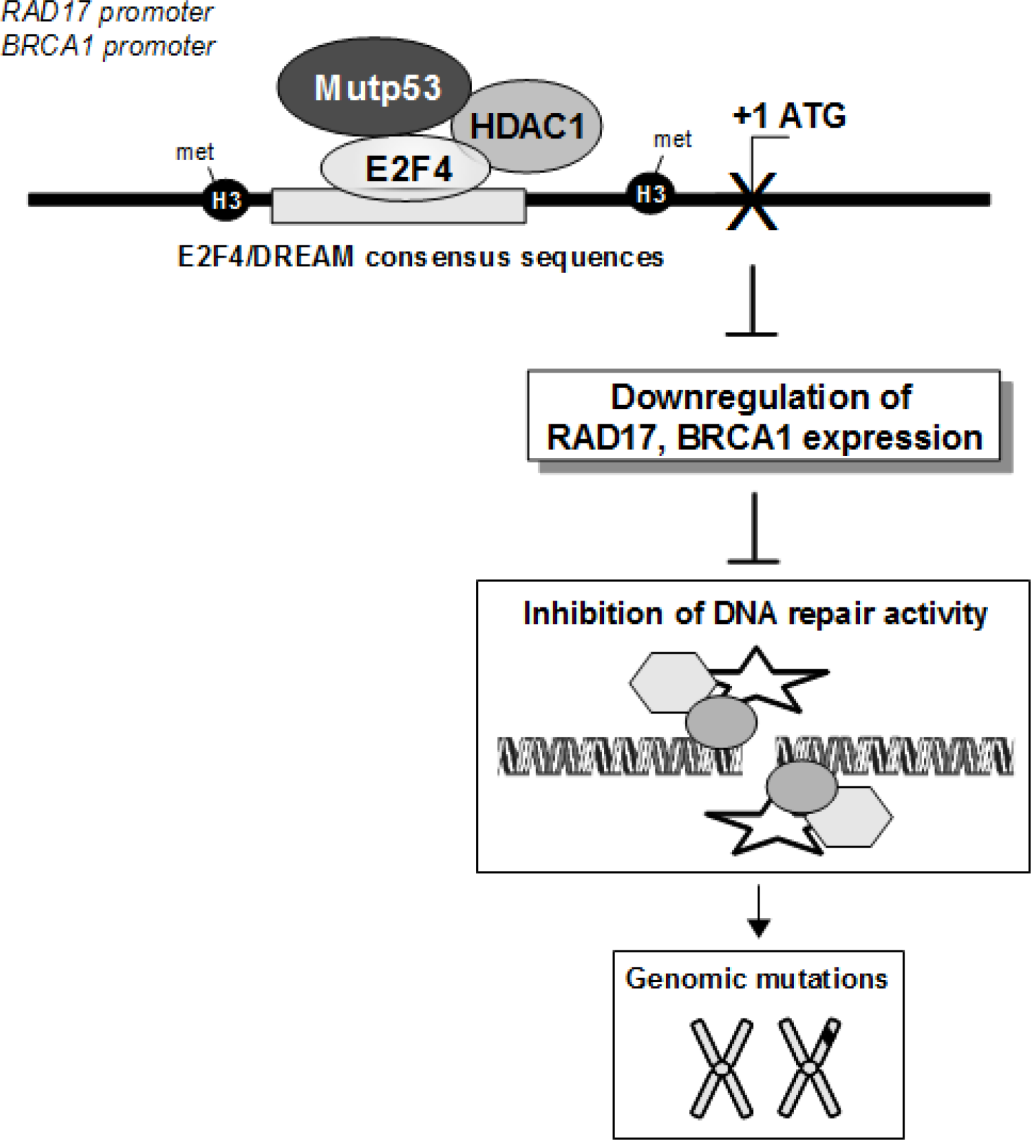
The depicted model proposes the molecular mechanisms underlying the transcriptional control exerted by mutp53/E2F4 repressive protein complex on BRCA1 and RAD17 gene expression. Its impact on DNA repair and tumorigenesis is also depicted

In this study, we explored the molecular mechanisms that underlie the inactivation of DNA repair genes in mutp53-expressing tumour cells. Here, we show that GOF mutp53 proteins are closely related to the intrinsic inability of tumour cells to repair DNA damage with the consequent accumulation of DNA mutations during cell growth (Figure 1). This underlines a direct role of mutp53 protein on DNA repair genes that might lead to increased IN. We show that mutp53 physically interacted with the transcription factor E2F4 that is a component of the DREAM repressor complex [44]. E2F4 plays an important role in the suppression of proliferation-associated genes, its gene mutation and increased expression are associated with human cancers [54]. Recent evidences show E2F4 may play an oncogenic rather than a tumor suppressor role in cells [55]. Our observations about epigenetic changes in the RAD17 and BRCA1 promoters due to the concomitant recruitment of mutp53 and E2F4 proteins provide additional insight to the growing role of E2Fs proteins in the de-regulation of DNA repair during tumoral cell proliferation, beyond their well-established roles in cell cycle checkpoint and the maintenance of quiescence. The protein complex mutp53/E2F4 is recruited onto E2F4 binding sites of RAD17 and BRCA1 promoters pairing with a global increase of histone H3 methylation and a decrease of histone H4 acetylation (Figures 3 and 4). This might contribute to chromatin transcriptional inactive status of RAD17 and BRCA1 promoter regions. To date, the transcriptional activity of mutp53 protein led mainly to the induction of its target genes through the cooperation with known transcription factors and histone acetyltransferases [9–13]. Together with the previously reported downregulation of CD95 (Fas/APO-1) gene by mutp53 protein [56], here we provide strong evidence that mutp53 can be a partner of transcriptional repressive protein complexes that lead to downregulation of the expression of *rad17* and *brca1* DNA repair genes. Previous studies showed that mutp53 proteins could interact with p73 and p63 thereby hampering their antitumoural effects through the displacement of both p73 and p63 from their specific binding sites within their target genes [16-19, 57]. Here, we originally report that GOF mutp53 proteins impair the antitumoural effects of tumour suppressor genes controlling their expression at the transcriptional level (Figure 8).

DNA damage is detected by “sensor” proteins such ATM and ATR that transmit the information to “transducer” proteins such as Chk1-2 kinases, which control the damage response through the phosphorylation of “effector” proteins such as 53 BP1, MRE11, MDC1, BRCA1-2 and RADs proteins [1, 26]. Defects in the DDR components such as p53, ATM, Chk2, BRCA1 and BRCA2 tumour suppressors contribute to the pathogenesis of many types of human cancers [1, 32, 33, 39, 40, 58-60]. Important studies described that DDR machinery is constitutively activated in early, premalignant lesions of major types of human solid tumours [39, 40].

Based on our findings that mutp53 protein suppresses repair activity we propose a novel mechanism of induction of IN which, at least for *brca1* and *rad17* genes, happens for the transcriptional repressive activity exerted by GOF mutp53 proteins (Figure 8). Since inhibitors of the aberrant kinase activity of DNA damage components are already used in cancer therapy, our data might contribute: a) to better define the molecular events underlying inefficient DNA repair in mutp53 tumour cells and consequently to tailor more accurately target specificity; b) to design therapeutic protocols that might combine kinase inhibitors with compounds interfering with mutp53 oncogenic activities.

## MATERIALS AND METHODS

### Cells and treatments

Lung cancer H1299 (p53 null), breast cancer SKBr3 (mutp53R175H), and MDA-MB-468 (mutp53R273H) cell lines were cultured in DMEM medium (Invitrogen, Carlsbad, CA, USA) and head & neck cancer CAL27 (mutp53H193L) cell line was cultured in RPMI medium (Invitrogen, Carlsbad, CA, USA), all media supplemented with 10% (v/v) FBS, penicillin and streptomycin (Invitrogen, Carlsbad, CA, USA).

### Plasmids and transfection

Mutant p53 exogenous expression was performed using pcDNA3-p53-R175H, pcDNA3-p53-R273H, pcDNA3HA-p53-D281G vectors and empty pcDNA3 was used as control [9, 20]. The pcDNA3-BRCA1 vector was kindly provided by Dr. M. Fanciulli. LUC-wtE2F-BRCA1 promoter and LUC-mutE2FA/B-BRCA1 promoter vectors are generously provided by Prof. P.M. Glazer. Cells were transfected with Lipofectamine 2000 by following the manufacturer's instructions (Invitrogen, Carlsbad, CA, USA).

### RNA interference

To transiently silence *TP53* expression we used siRNA oligonucleotides targeting p53 in the aa 245–251 sequence [9] and commercial siRNA smart pool of three oligonucleotides (*si-p53 sp* in the manuscript) transiently targeting p53 and E2F4 in their transcripts and the siRNA oligogonucleotides for BRCA1 and RAD17 knock-down were provided by Santa Cruz Biotech. (Santa Cruz Biotech., Santa Cruz, CA, USA). The sequence of si-GFP employed as nonsilencing control was 5′-GGCTACGTCCAggaGCGCACC-3′.

### Cell extracts, immunoprecipitations and protein blotting

Cells were homogenized in a lysis buffer composed by 50 mM Hepes pH 7.5, 5 mM EDTA pH 8.0, 10 mM MgCl2, 150 mM NaCl, 50 mM NaF, 20 mM β-glicerophosphate, 0.5% NP40, 0.1 mM sodium orthovanadate, 1 mM PMSF, 1 mM DTT and protease and phosphatase inhibitors. Extracts were sonicated for 20 min and clarified by centrifugation to remove cell debris. To homogenize the cells destinated to the the co-immunoprecipitation of endogenous mutp53 and E2F4 proteins (Figures 5C and D), we have modified the lysis buffer with 150 mM Hepes pH 7.5, 300 mM NaCl and 1% Triton-X100. In the co-immunoprecipitation the samples were diluited with PBS 1X at 1:1. Protein concentrations were determined by colorimetric assay (Bio-Rad, Hercules, CA, USA). Western blotting was performed using the following primary antibodies: mouse monoclonal p53 (DO1), GAPDH (Santa Cruz Biotech.); rabbit polyclonal RAD17, E2F4, p53 (Santa Cruz Biotech.); rabbit polyclonal BRCA1, Actin, CHK1, P-H2AX (Ser139) (Cell Signaling Tech., Danvers, MA, USA). Immunostained bands were detected by chemiluminescent method (Thermo Fisher Scientific, Rockford, IL, USA). For each immunoprecipitation, 1 μg of rabbit E2F4 antibody (Santa Cruz Biotech.) and 1 μg of rabbit IgG (Santa Cruz Biotech.) as control were used. Precleared extracts were incubated with protein A/G-Agarose beads (Thermo Fisher Scientific, Rockford, IL, USA) in lysis buffer containing 0.05% BSA and antibodies, under constant shaking at 4°C for 3hours. After incubation, agarose bead-bound immunocomplexes were rinsed with lysis buffer and eluted in 50 ml of SDS sample buffer for western blotting. Western blot analysis was performed with the aid of the enhanced chemiluminescence system (Thermo Fisher Scientific, Rockford, IL, USA).

### RNA isolation, quantitative real-time PCR analysis

Total RNA was extracted from cells by using TRI Reagent (Invitrogen, Carlsbad, CA, USA) in accordance with manufacturer's instructions. Five micrograms of total RNA were reverse-transcribed at 37°C for 60 min in the presence of random hexamers and Moloney murine leukemia virus reverse transcriptase (Invitrogen). PCR analyses were carried out using oligonucleotides specific for the genes listed in Supplementary Table 1. Transcripts were measured by real-time PCR using the SYBR Green assay (Applied Biosystems, Carlsbad, CA, USA) with a StepOne instrument (Applied Biosystems). BRCA1 primer sequences were taken from Mullany et al. [34]. The other primers were designed with Primer3 version 0.4.0 (http://frodo.wi.mit.edu/primer3/). All primer sets worked under identical quantitative PCR cycling conditions with similar efficiencies to obtain simultaneous amplification in the same run. The 2^−ΔΔCT^ method for relative quantitation of gene expression was used to determine mRNA expression levels. *GAPDH* and *β–actin* gene expression was used as endogenous controls to standardize mRNA expression. All reactions were performed in duplicate. P-values were calculated with two-tailed t-test. Statistically significant results were referred with a p-value < 0.05.

### RAPD assay

100 ng of genomic DNA from cell lines, was amplified by PCR as previously described [33] and using the following primers: #1: 5′-CCGGCTACGG-3′; #2: 5′-CAGGCCCTTC-3′; #3: 5′-AA CGGTCACG-3′; #4: 5′-AGCTGCCGGG-3′; #6: 5′-GGTCTGAACC-3′; #7: 5′-AAGGCTAACG-3′. The PCR reactions were performed with AmpliTaq Gold® PCR Master Mix (Invitrogen, Carlsbad, CA, USA) following the manufacturer's instructions. PCR products were run onto a 2% agarose gel and stained with ethidium bromide. The size of the PCR products was identified using O' GeneRuler DNA ladder mix (n° SM1173; Fermentas). Control PCRs to check the quality and the amount of genomic DNA were performed using the oligogonucleotides that amplify 280 bp on genomic DNA described in “Primers used in PCR of ChIP experiments” as “negative region” in the Supplementary Table 2.

### Comet assay

To quantify the DNA damage the single cell gel electrophoresis was performed using Comet Assay kit and following the manufacturer's instructions (Trevigen Gaithersburg, MD, USA). Cells were detached with trypsin and embedded in 1% low melting agarose and spun onto microscopy slides coated with 1% Agarose. Cells were lysed in the alkaline lysis solution and then run in running solution (300 mM NaOH, 1 mM EDTA) for 30 min at 1V/cm and about 300 mA. DNA was dried with 70% ethanol and stained with DAPI and mounted with Vectashield (Vector Labs, Burlingame, CA, USA). Pictures were taken using an Axiovert 200 M microscope and Axiovision acquisition program (Zeiss). At least 100 cells were scored for each slide. To evaluate the assays, it was used a software tool providing automated analysis of comet assay images (OpenCOMET; http://www.opencomet.org) [38]. The extent of DNA damage is related to the amount of DNA in the tail. The percentage of DNA in the tail is plotted in the lower panels of each figures.

### T4 DNA ligase *in vitro* assay

T4 DNA ligase *in vitro* assay was performed as previously described [37]. Linearized pUC19 DNA vector (Takara biotechnology, Dalia, CO., LTD) was prepared as previously described. 200 ng of this DNA was incubated with nuclear extracts for 1h at 25°C in a reaction mixture containing 1 × ligase buffer and 1 μl of T4 DNA ligase (200 U, New England Biolabs). Reactions were impeded and de-proteinated by adding Proteinase K enzyme (Invitrogen) followed by 15 min incubation at 37°C. DNA ligation products were recovered by extraction with phenol:chloroform (1:1 v/v) and ethanol precipitation and separated by 1% agarose gel electrophoresis. The gel was visualized by staining with ethidium bromide and represented as an inverted image. The size of the DNA products was identified using O' GeneRuler DNA ladder mix (n° SM1173; Fermentas).

### DNA repair *in vitro* assay

DNA repair *in vitro* assay was performed as previously described [62]. Linearized pUC19 plasmid DNA was prepared by digestion with *Hin*dIII restriction enzyme to produce complementary ends. DNA was purified from agarose gel with a DNA extraction kit (Qiagen). Cells were allowed to swell for 10 min on ice in a hypotonic buffer (10 mM HEPES-KOH pH 7.9; 1.5 mM MgCl2; 10 mM KCl; 0.5 mM dithiothreitol; 0.2 mM phenylmethylsulfonyl fluoride) and subjected to three cycles of freeze-thawing. Cell-extracts were clarified by centrifugation at 13000rpm. The repair reactions were carried out in a total volume of 50 μl, containing 50 mM Tris, pH 8.0, 5 mM MgCl2, 1 mM ATP, 1 mM DTT, 5% polyethyleneglycol 8000, a protease inhibitor cocktail, 200 ng of substrate DNA and 20 μg of proteins, and incubated at 17°C for 16 h. Repair was stopped by adding 0.4% SDS and incubation at 65°C for 15 min. DNA was recovered by extraction with phenol:chloroform (1:1 v/v) and ethanol precipitation and repair products were identified by 1% agarose electrophoresis. The gel was visualized by staining with ethidium bromide and represented as an inverted image. The size of the DNA products were identified using O' GeneRuler DNA ladder mix (n° SM1173; Fermentas).

### ChIP and Re-ChIP experiments

1% formaldehyde cross-linking and chromatin immunoprecipitations were performed as described [9, 20]. The chromatin solution was immunoprecipitated with sheep anti-p53 Ab7 (Millipore, Billerica, MA, USA), rabbit anti-E2F4 (Santa Cruz Biotech.), rabbit H4Ac and rabbit H3K9Met3 (Cell Signaling Tech., Inc.) or no antibody as negative control. The immunoprecipitations were performed using Pierce ChIP-grade Protein A/G magnetic beads (Thermo Fisher Scientific, Rockford, IL, USA). Primers used for the amplification of the different regulatory regions are listed in the Supplementary Table 2. E2F4 Region 2 consensus sequence on BRCA1 promoter was selected from the literature and E2F4 Region 1 consensus sequence was identified by MatInspector software (http://www.genomatix.de) [44]. We provided the Supplemental File 1 that reports 4000 bp upstream of First Exon of hBRCA1 promoter with the E2F4 consensus sequences highlighted. E2F4 consensus sequences on RAD17 promoter were identified using MatInspector software (http://www.genomatix.de). The TSS of RAD17 and BRCA1 genes was found by Eponine software prediction [63].

In the Re-ChIP experiments the chromatin was eluted with 10 mM DTT at room temperature for 30 min. The eluted chromatin was diluted 10-fold with re-chIP buffer (15 mM Tris-Cl pH 7.5, 1% Triton X-100, 150 mM NaCl and 1 mM EDTA) and the second-round of ChIP was carried out according to the regular ChIP procedure. The promoter occupancy was analyzed by qRT-PCR using the SYBR Green assay (Applied Biosystems, Carlsbad, CA, USA) and the 7500 Fast Real-Time PCR System (Applied Biosystems). P-values were calculated with two-tailed t-test. Normalization was performed to the amount of input chromatin. Statistically significant results were referred with p-value < 0.05.

### Head and neck tumour tissue samples

The casuistry of Head and Neck squamous cell carcinoma patients was previously described in Ganci et al. [45, 46]. Briefly, patients with primary HNSCC and no previous treatment with radiotherapy or chemotherapy who underwent resection in the Otolaryngology Head and Neck Surgery Department were considered in this study. The study (protocol CE/379/08) was approved by the Scientific Ethic Committee of the Regina Elena Italian Cancer Institute in Rome. TP53 mutations in these samples were evaluated by sequencing of exons 2 though 11 of TP53 gene. BRCA1 and RAD17 mRNAs were analyzed by RT-qPCR on a group of 63 HNSCC tumor samples and on their normal counterparts. Normal samples were collected from surgery resection margin for each patient and were all histologically checked for the absence of tumor cells (described in Ganci et al, 2013). Normal samples were also subjected to TP53 sequencing and resulted negative for mutations.

### mRNA expression data

We used the publicly available data sets: GSE27157 [39] and GSE31812 [40]. The mRNA expression data were measured using Affymetrix Human Genome U133 Plus 2.0 and Human Gene 1.0 ST arrays respectively.

### Statistical methods

Statistical analysis was done using two-sided Student's *t*-test. Differences were deemed statistically significant at *P* < 0.05.

## SUPPLEMENTARY MATERIALS, FIGURES AND TABLE



## ABBREVIATIONS

GOF: gain-of-function
IN: genomic instability
mutp53: mutant p53 protein
HR: homologous recombination
NHEJ: non-homologous end joining
DSBs: double strand breaks
HNSCC: head and neck squamous cell carcinoma
