# Supplementary material for: Gain of function mutant p53 proteins cooperate with E2F4 to transcriptionally downregulate RAD17 and BRCA1 gene expression
Source: Oncotarget. 2015 Feb 5;6(8):5547–66. doi: 10.18632/oncotarget.2587 (PMC4467386; doi:10.18632/oncotarget.2587)
Supplement: Supplementary file 1 [file oncotarget-06-5547-s001.pdf]

SUPPLEMENTARY MATERIALS, FIGURES AND TABLE

Cells

MCF7 (wtp53) cell lines were cultured in DMEM medium (Invitrogen, Invitrogen, Carlsbad, CA, USA) supplemented with 10% (v/v) FBS (Invitrogen, Invitrogen, Carlsbad, CA, USA).

GST recombinant protein

Recombinant proteins employed in the DNA repair assay (Supplemental Figure 3D) were produced and purified as previously described (Strano et al., 2000; Valenti *et al.*, 2011). Briefly, *Escherichia coli* cells (BL21-DE3) were transformed with the appropriate plasmid grown at 37°C in LB medium to an optical density (600 nm) of 0.4, and induced with 0.5 mM isopropyl-1-thio-galactopyranoside for 3 h at the same temperature. Cells were harvested by centrifugation and lysed in ice cold phosphate-buffered saline (PBS) containing 0.1% Triton X-100, 0,5% NP40, 1 mM dithiothreitol (DTT), protease inhibitors, by probe sonication. After centrifugation at 12,000 *g*, supernatant fractions were incubated with either glutathione-Sepharose beads (G 4510; Sigma-Aldrich, St. Louis, MO), for 1 h at 4°C under constant shaking. After washes in PBS, GST-fusions were eluted with either 100 mM Tris-HCl, pH 8, 250 mM NaCl containing 10 mM glutathione (G 4251; Sigma-Aldrich).

Luciferase transactivation promoter assays

BRCA1 promoter luciferase assays and the NHEJ Luciferase assays were performed as described previously [43]. 2×10^5 cells were plated into 12-well culture plates and transfected with 100pmol of each siRNA and 100 ng of each reporter construct using Lipofectamine 2000 (Invitrogen, Carlsbad, CA, USA) according to the manufacturer's specifications. Firefly luciferase activity was measured using a Luciferase Reporter Assay System kit (Promega Corporation) according to the manufacturer's instructions. Luciferase activity was normalized to total protein and assayed in triplicate.

Kaplan-Meier analysis

Gene expression dataset raw data and survival information of 478 BCL patients were downloaded from GEO (http://www.ncbi.nlm.nih.gov/geo/) and the Kaplan-Meier analysis was performed by using the on-line tool www.kmplot.com [53], where eight public datasets were considered. Gene expression profiles were performed by using Affymetrix HG-U133A (GPL96) and HG-U133 Plus 2.0 (GPL570) microarrays were only considered. The latter are the most frequently used and have 22, 277 probe sets in common. To analyze the prognostic value of genes, the cohorts were divided into two groups according to the median (or upper/lower quartile) expression of the gene. The two groups were compared in terms of relapse free survival (RFS). A survival curve was displayed, and the hazard ratio with 95% confidence intervals and logrank *P* value are calculated. *P* values < 0.05 were considered statistically significant.

Supporting information table S1

Primer sequences for amplification of cDNAs in Real Time PCR experiments.

Supporting information table S2

Primers used for the amplification of the different regulatory regions of BRCA1 and RAD17 promoters. Oligonucleotides for the *Negative Region* amplifying a fragment encompassing 68509119-68509420 bp sequence of cyclin B1 gene were designed on a sequence of human cyclinB1 gene where mut p53 was not recruited as previously demonstrated (Di Agostino *et al*., 2006, Figure S3 Supplemental Data).

Supporting information table S3

Mutational status of *TP53* gene of HNSCC tumors used for RT-qPCR expression profiles.

Supporting information table S4

Fisher's exact test was used to examine the significance of the association between clinical variables and status of p53 mutation. Fold <1 means down-regulation and fold >1 means up-regulation.

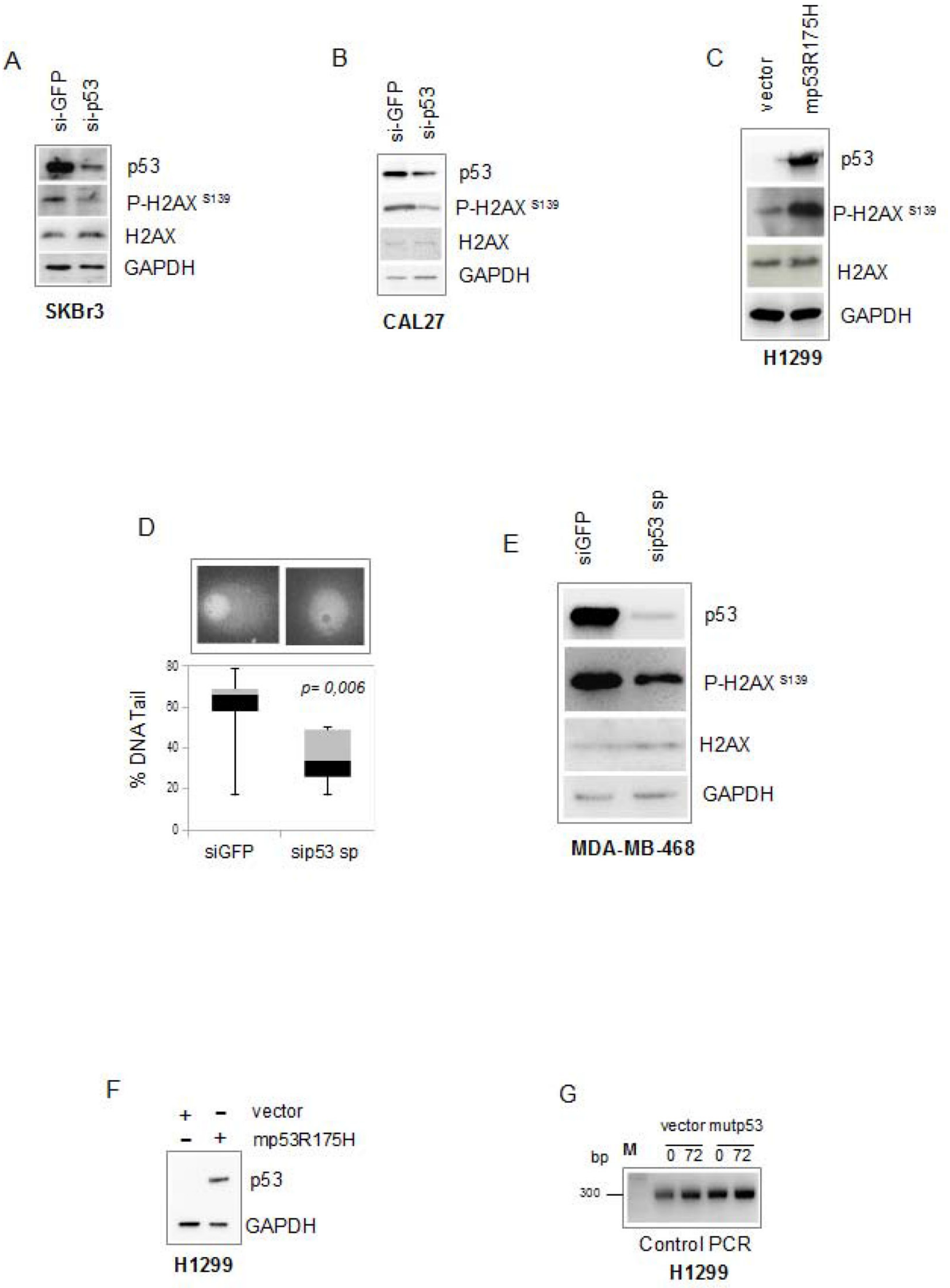

Supplementary Figure S1: (A-C) Western blot analysis was performed from 30 µg of whole protein lysates of SKBr3 (a) and CAL27 (b) cells knocked-down for p53 with si-p53 and with si-GFP oligonucleotides as control, and in H1299 cells transfected with pcDNA3-p53R175H vector and empty pcDNA3 as control (c). The probing was carried out with the indicated antibodies. A pool from these cells was employied in the comet assays described in the Figures 1A-C. **(D)** MDA-MB-468 cells transfected with siGFP and with sip53 sp siRNAs (siRNA smart pool of three oligonucleotides by Santa Cruz Biotech.) were analyzed for amount of DNA damaged by comet assay. Representative pictures are shown in the upper panel. It was used a software tool to provide an automated analysis of comet assay images (OpenCOMET; www.opencomet.org). The extent of DNA damage is related to the amount of DNA in the tail. The percentage of DNA in the tail is plotted in the lower panels of each figures. About 100 cells were evaluated for each sample. *P*-values were calculated with two-tailed t-test. Statistically significant results were with *p*-value < 0.05. **(E)** Western blot analysis was performed in MDA-MB-468 cell line knocked-down for p53 with si-p53 sp and with si-GFP as control. The probing was carried out with the indicated antibodies. **(F and G)** Protein extracts from H1299 cell line (f) transfected with pcDNA3-p53R175H vector and empty pcDNA3 as control were analyzed for p53 protein expression by western blotting. The genomic DNA (g) extracted from an aliquot of these cells was tested in control PCRs using the oligogonucleotides that amplify 280 bp on genomic DNA described in "Primers used in PCR of ChIP experiments" as "negative region" in the Supplementary Table 2. These genomic preparations were used as template in the RAPD-PCR analysis described in Figure 1D.

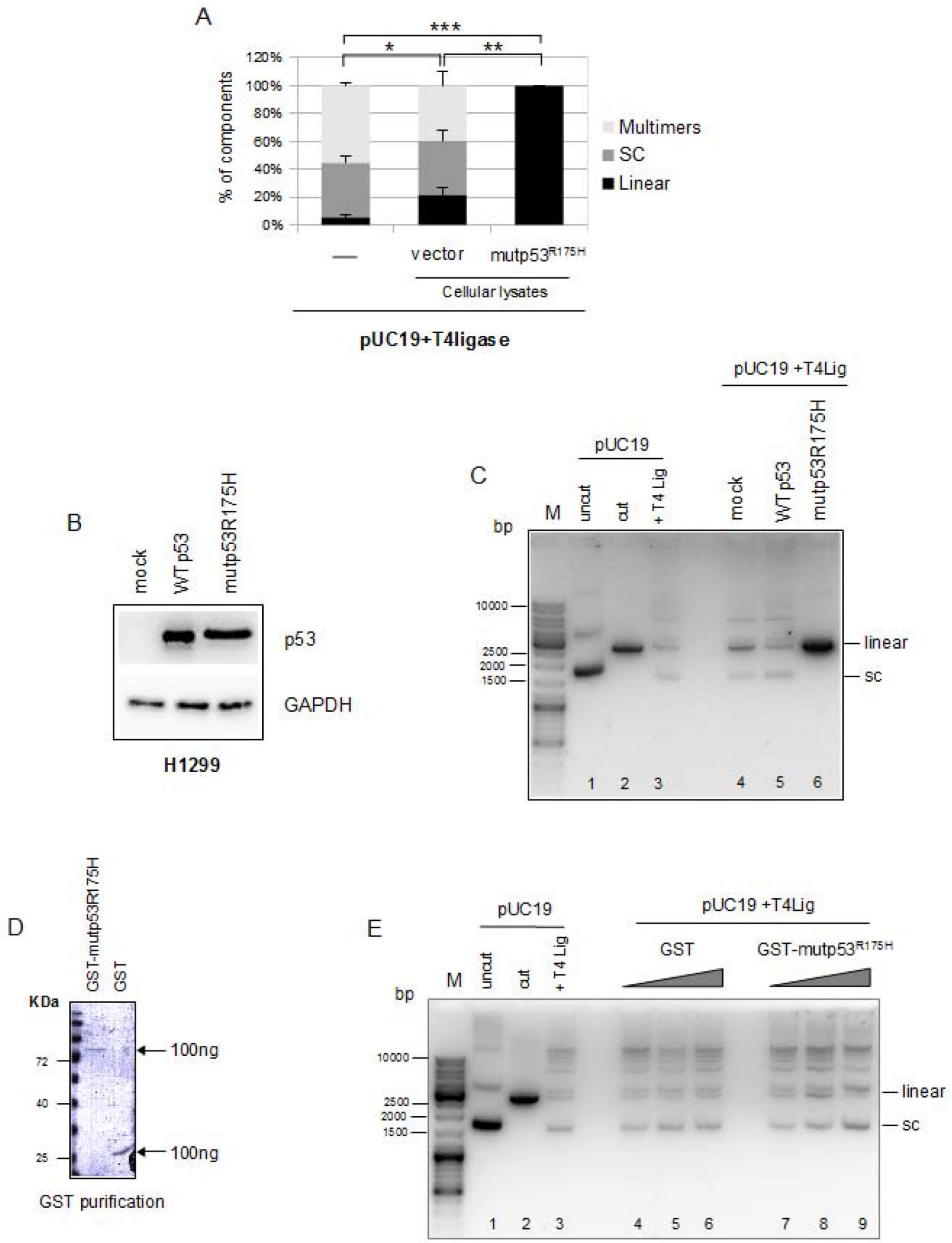

Supplementary Figure S2: (A) Densitometric analysis (Image J software; http://imagej.nih.gov/ij/) from all the experiments of T4 ligase DNA repair assay from H1299 cells transiently transfected with pCDNA3-vector and pCDNA3-mutp53R175H presented in the manuscript (and others not shown). The cellular lysates provoked a different pattern of bands than the sample with only T4 ligase and cut vector. Linear (linearized pUC19 vector), SC (supercoiled-coil re-ligated vector), Multimers (re-ligated vector in different ways). The *p*-values are referring only to the linear component in black columns (* = 0,007; ** = 3×10^{-6}; *** = 2×10^{-8}; $n = 6$). **(B)** Protein extracts from H1299 cell line transfected with pcDNA3-wild-type p53, pcDNA3-p53R175H vectors and empty pcDNA3 as control were analyzed for p53 protein expression by western blotting. **(C)** Comparison of ligation products of 5′-cohesive-ended linear DNA (lane 2) in the presence of T4 DNA ligase alone (lane 3) or following pre-incubation with whole protein extracts of H1299 cells transfected with wild type p53 (lane 5), mutp53R175H (lane 6) or control expressing vectors (lane 4). Numbers indicate the lanes. **(D)** 100ng of GST-mutp53R175H and GST alone purified by glutathione-Sepharose beads have run on 10% acrylamide gel. **(E)** Comparison of ligation products of 5′-cohesive-ended linear DNA (lane 2) in the presence of T4 DNA ligase alone (lane 3) or following incubation with increasing amount of GST (100, 200 and 500ng; lanes 4, 5 and 6 respectively) or GST-mutp53R175H (100, 200 and 500ng; lanes 7, 8 and 9 respectively) proteins. Numbers indicate the lanes.

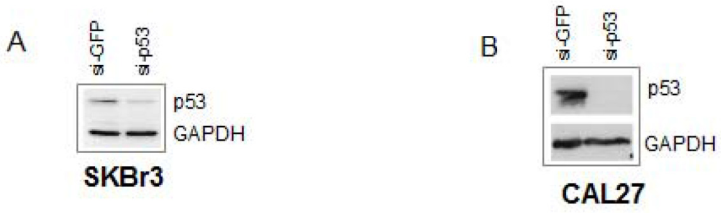

Supplementary Figure S3: (A and **B)** Western blot analysis was performed from 30 µg of whole protein lysates of SKBr3 (a) and CAL27 (b) cells knocked-down for p53 with si-p53 and with si-GFP oligonucleotides as control. A fraction of these lysates were used in the DNA repair assays described in the Figures 1D and E, respectively.

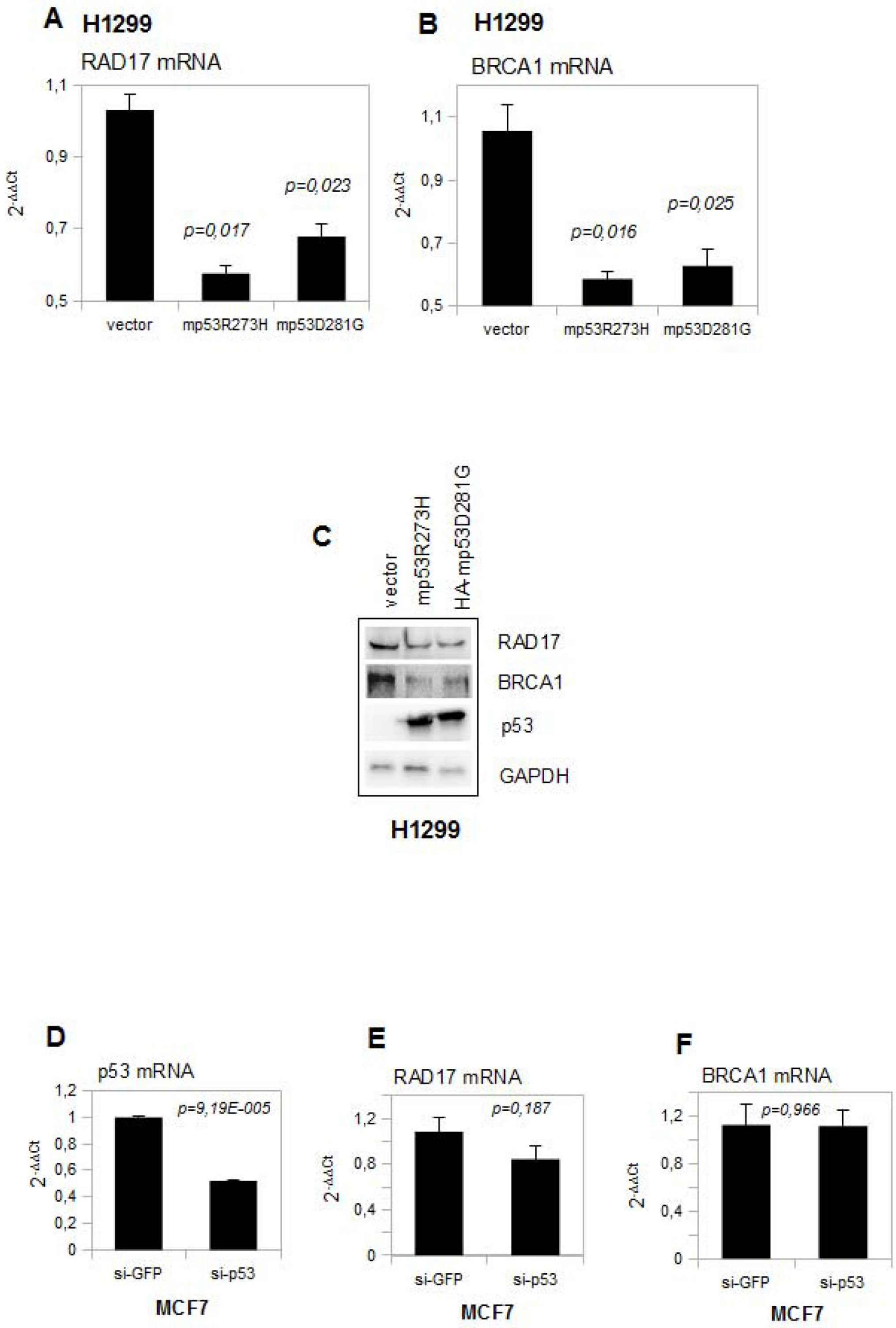

Supplementary Figure S4: **(A** and **B)** H1299 cells were transfected with 2 µg of pcDNA3-p53R273H and pcDNA3HA-p53D281G and from the cDNA obtained by these cells was performed a RT-qPCR to evaluate the RAD17 (a) and BRCA1 (b) mRNAs expression. **(C)** Western blot analysis was performed in H1299 cell line transfected as previously described. The probing was carried out with the indicated antibodies. **(D-F)** RT-qPCR analysis of *p53, RAD17* and *BRCA1* expression, respectively, in breast MCF7 cell line transfected with si-GFP and si-p53 siRNAs. The experiments were produced in biological triplicates. *P*-values of the shown qRT-PCR experiments were calculated with two-tailed t-test. Statistically significant results were with *p*-value < 0.05.

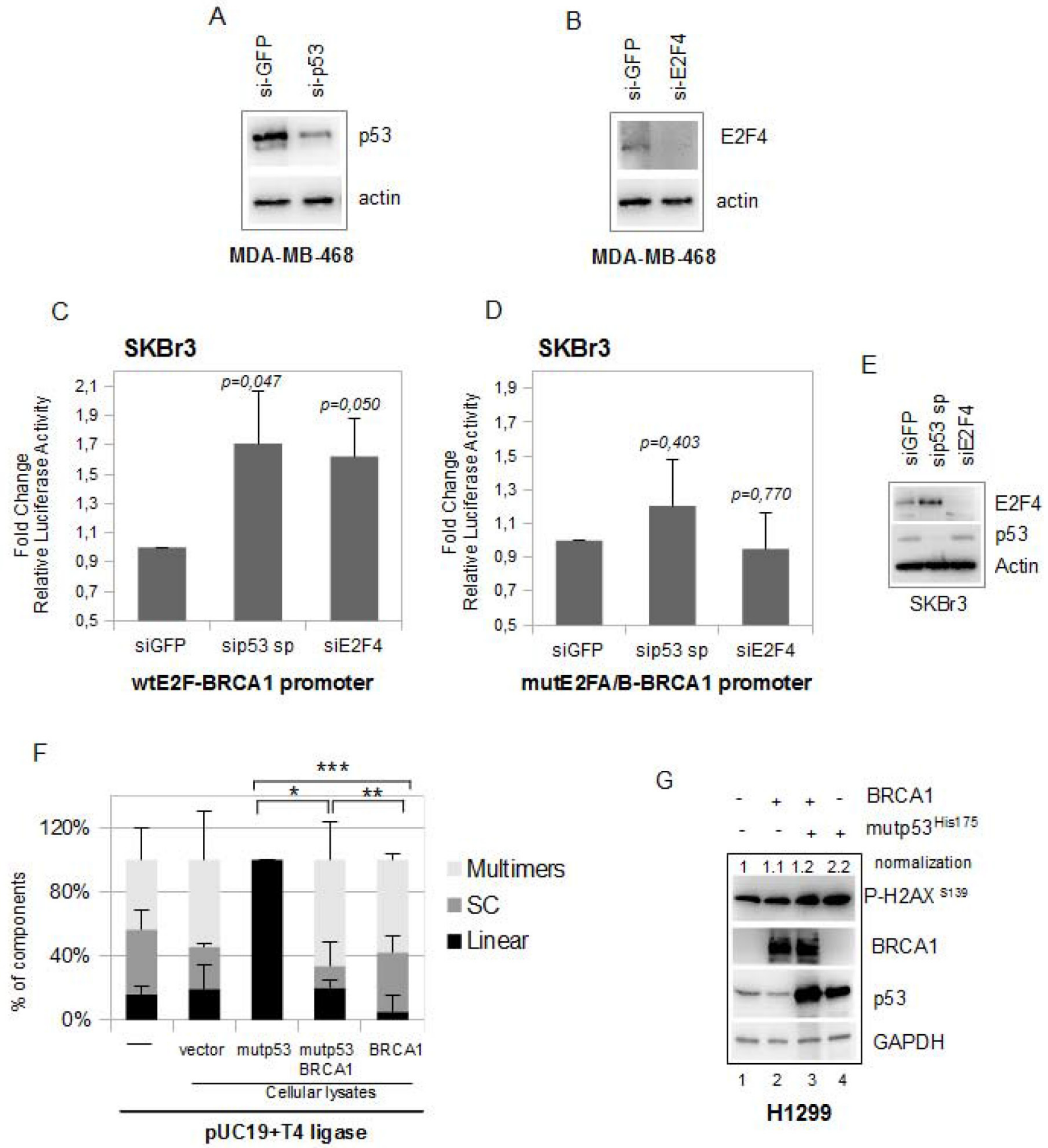

Supplementary Figure S5: **(A** and **B)** Western Blot analysis for the expression of the indicated gene products from MDA-MB-468 cells respectively transfected with si-p53 (a), si-E2F4 (b) and si-GFP as control. The cross-linked chromatin from these cells were used in the ChIP and Re-ChIP analysis described in the Figures 3 and 4. **(C** and **D)** SKBr3 cells interfered with siGFP, sip53 sp and siE2F4 oligos for 18 h (over night), were further transfected with wtE2F-BRCA1 (c) and mutE2FA/B-BRCA1 (d) and Firefly luciferase gene reporter constructs (100 ng). 24 hours after the second transfection the cells were harvested for the evaluation of luciferase activity. The shown data represent the mean +SD of triplicate determinations from three independent experiments. The histograms represented the folds over the siGFP control. **(E)** SKBR3 cells transfected with siGFP, sip53 sp and siE2F4 oligos used in the transactivation LUC assays showed in (c) and (d) were analyzed by western blotting at the end of the experiments. **(F)** Densitometric analysis (Image J software; http://imagej.nih.gov/ij/) from three experiments of T4 ligase DNA repair assay from H1299 cells transiently transfected with pCDNA3-vector and pCDNA3-mutp53R175H and BRCA1 in different combination as described in Figure 6A of the paper. The cellular lysates provoked a different pattern of bands than the sample with only T4 ligase and cut vector. Linear (linearized pUC19 vector), SC (supercoiled-coil re-ligated vector), Multimers (re-ligated vector in different ways). The *p*-values are referring only to the linear component in black columns ($* = 10^{-5}$; $** = 0{,}008$; $*** = 10^{-7}$; $n = 3$). **(G)** 50 µg of protein extract from H1299 cell line transfected as described in (f) was analyzed by western blotting with the indicated antibodies.

(*Continued*)

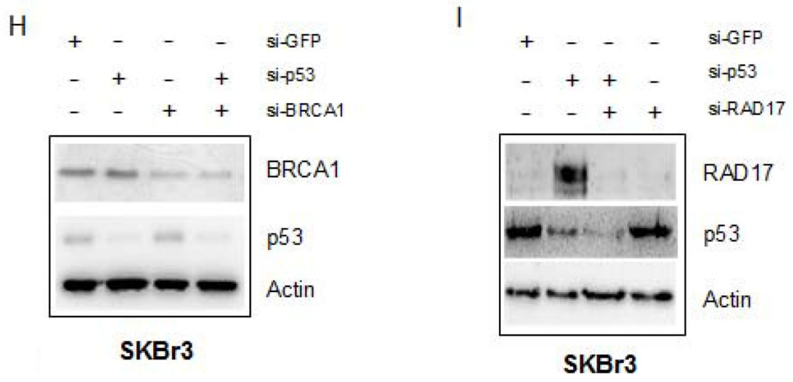

Supplementary Figure S5 (*Continued*): (H and I) SKBr3 cells were transiently transfected with ApaI-linearized vector, to perform the luciferase assay shown in Figures 6D and E, and with either siBRCA1 (h) and siRAD17 (i) oligos. After 48 h the cells were harvested and the functional changes in NHEJ were assessed measuring the Firefly Luciferase activity. 20 µg of protein extract from these cells were analyzed by western blotting with the indicated antibodies.

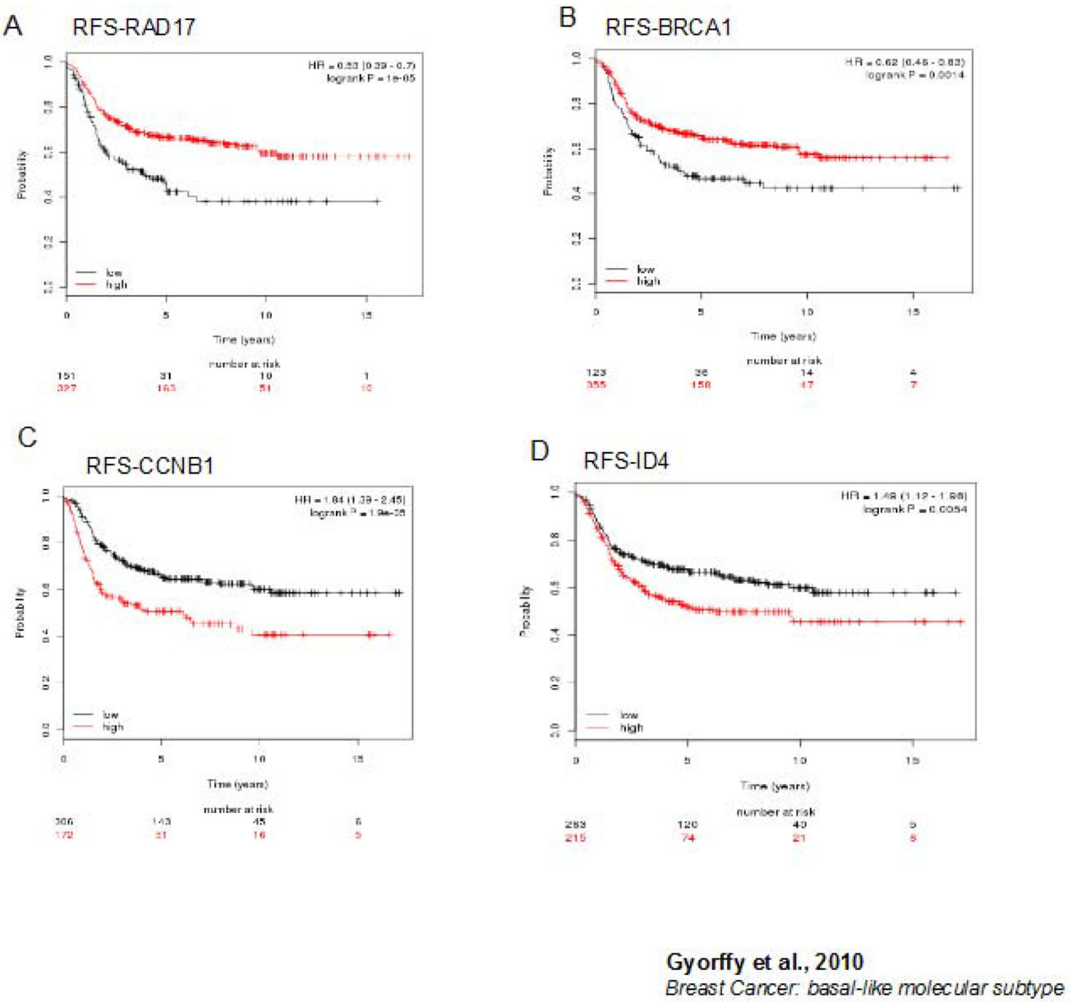

Supplementary Figure S6: (A-C) Kaplan-Meier survival curves of relapse free survival (RFS) of BCL breast cancer patients classified according to the expression of RAD17 (a), BRCA1 (b), CNNB1 (c) and ID4 **(D)** mutp53-target genes. Statistically significant results with *p*-value < 0.05, $N = 478$). The two compared groups are the patients with the highest expression (red) levels of each gene versus the patients with the lowest expression (black).

Supplementary Table S1. Primers used for qRT-PCR experiments

**Rad17_a FW**	5′- GGAGCATGGTATTCAAGTACAAG -3′
**Rad17_a RV**	5′- GGGAAACATATGGAAGCTTGA -3′
**Rad17_b FW**	5′- TGAGAGCCAGTGAATTTCTGAG -3′
**Rad17_b RV**	5′- TGCATCACACCTCTCGTAGC -3′
**BRCA1 FW (Mullany et al., 2012)**	5′- TCCCATCTGTCTGGAGTTGA -3′
**BRCA1 RV (Mullany et al., 2012)**	5′- TGTGAAGGCCCTTTCTTCTG -3′
**CHK1 FW**	5′- TTCTATGGTCACAGGAGAGAAGG -3′
**CHK1 RV**	5′- ATAAACCACCCCTGCCATGA -3′
**CCNB1 FW**	5′- GTTCCTACGGCCCCTGCT -3′
**CCNB1 RV**	5′- ATttTGGCCTGCAGTTGTTC -3′
**GAPDH FW**	5′- GAGTCAACGGATTTGGTCGT -3′
**GAPDH RV**	5′- GAGTCAACGGATTTGGTCGT -3′
**β–Actin FW**	5′- GAGGCCCAGAGCAAGAGAG -3′
**β–Actin RV**	5′- AGGTGTGGTGCCAGATTTTC -3′

Supplementary Table S2. Primers used in PCR of ChIP experiments: BRCA1 promoter

**BRCA1 promoter**	
E2F4_Reg1 FW	5′- CTAACATGGCGGACAAAGACA -3′
E2F4_Reg1 RV	5′- CAATGGGGTGGTCGTTTT -3′
E2F4_Reg2 FW	5′- TTCTGAGAGGCTGCTGCTTA -3′
E2F4_Reg2 RV	5′- CCCGTCCAGGAAGTCTCAG -3′
**RAD17 promoter**	
E2F4_Reg1 FW	5′- CAGGAGAATCCCTTGAACCA -3′
E2F4_Reg1 RV	5′- TGATTCGAGGTGGCTAATTTTT -3′
E2F4_Reg2 FW	5′- CTACCGGGAAGCAGATATGG -3′
E2F4_Reg2 RV	5′- GCTTGGCAACCTGCTTAGTC -3′
**Negative Region**	
Negative_Reg FW	5′- AAGTATGCCACATCGAAGCA-3′
Negative_Reg RV	5′-AAGGGGCCACAAGCTTTATT-3′

Supplemental Table 3.

Patient number	Codon	Type of mutation
′5′	H193Y	missense
′18′	V157I, R158H	missense
′69′	N247in frame	framshift
′84′	F106 in frame	framshift
′105′	H179R	missense
′93′	del. 4 bp introne 8	frameshift
′3′	wt	
′87′	wt	
′90′	wt	
′15′	wt	
′45′	del. 3 bp exon 9	frameshift
′12′	wt	
′115′	wt	
′109′	C275R	missense
′97′	wt	
′120′	wt	
′71′	wt	
′101′	R213X	nonsense
′86′	I255F	missense
′104′	H179R	missense
′99′	E198X	nonsense
′28′	P278T	missense
′29′	R156H;C275S;R282Q	missense
′49′	E198X	nonsense
′17′	R196X	nonsense
′61′	V157T	missense
′82′	del 2 bp P152-E153 not in frame	frameshift
′70′	S183X	nonsense
′74′	slice site exon 9	splice site
′42′	wt	
′8′	wt	
′7′	G266R	missense
′66′	del 1 bp exon 4 not in frame	frameshift
′113′	R196X	nonsense
′63′	P273C	missense
′88′	del 1 bp exon 8	frameshift
′54′	wt	

(*Continued*)

Patient number	Codon	Type of mutation
′48′	wt	
′14′	P278S	missense
′37′	W146X	nonsense
′22′	R248Q	missense
′59′	H193R	missense
′58′	ins 1 bp not in frame	frameshift
′51′	Y234C	missense
′55′	Y205C	missense
′36′	wt	
′107′	wt	
′57′	wt	
′102′	wt	
′10′	wt	
′1′	wt	
′2′	wt	
′40′	wt	
′44′	wt	
′41′	wt	
′112′	wt	
′73′	wt	
′32′	wt	
′65′	wt	
′60′	wt	
′114′	wt	
′67′	wt	
′33′	wt	

Supplemental Table S4.

	WT p53	MUT p53	Fisher's exact test
**Total patients**	**#**<br>**31**	**#**<br>**32**	**pval**<br>**-**
Treated\untreated\unknown	15\15\1	24\8	0.07
G1G2\G3\unknown	38\23	16\14\2	0.19
N+\N0\unknown	10\20\1	17\14\1	0.12
T1T2\T3T4\unknown	20\11	12\19\1	0.074
Tobacco Yes\No	10\21	5\27	0.15
Alchol Yes\No	18\13	11\23	0.08
Oral cavity\larynx pharynx	19\12	18\14	0.8
Fold RAD17 < 1\Fold RAD17 > 1	14\30	17\2	0,0001
Fold BRCA1 < 1\Fold BRCA1 > 1	11\24	20\8	0,002

SUPPLEMENTARY FILE 1

4000 bp upstream of First Exon of hBRCA1 promoter (Downloaded by Genome Browser-UCSC)

>hg19_knownGene_uc010whn.2 range = chr17:41196312-41281500 5′pad = 0 3′pad = 0 strand = - repeatMasking = none

EXONS in UPPER cases

Introns, 3′UTR and 5′UTR in lower cases

Highlighted in yellow E2F4 consensus binding sequences identified by us using MATInspector. *MatInspector* is Genomatix' internationally renowned program for the identification of transcription factor binding sites (www.genomatix.de.

In red, E2F4 consensus sequences, E2FA(+) and E2FB(+)respectively, described in the paper of Bindra et al. 2005 Cancer Res. Figure 2A

gctggaccatatccaggtctccataaggaactctctttggctggcatgta
aaaaaattctttcagcaagagcattaatcttgaacgtgtgtaaacacgaa
agctatggttaaccctacctgctccttaggtctacagccagtagagtcag
atagctgggtcctaggccttcagctttcataattgacattattgttaagt
ccatgtgactgactcaccttgtcttggcacaagttagcttttcctccaca
tcgattttttttctttcagcctcaacaaaatccctttaatgaaacaaac
ctggcaacccgaactttggttactgtctccaccagaggcctgtacaagcc
aagttatccaagctgaggccgggagtggtggctcatgcctgtaatcccag
cactttgggaggccaaggcaggaggatcgcttgagctcaggagttcaaga
ccagcctgggctggtctttgagaaagcgagactttgtctcgaaaaaaaaa
aaaaaagtgtggcctgaaagttgagtgtttgacaaaaaggagaaacagtg
cctttccaaaaccaaaggtccctaaccaagatctctttaacggggtctcg
aaaaaaggagaatgggatgagaaggatatatgggtagtgtcatttttttaa
cttgcagatttcatcctagtcttccagttatcgtttcctagcactccatg
ttcccaagatagtgtcaccaccccaaggactctctctcattttctttgcc
tgggccctctttctactgaggagtcgtggccttccatcagtagaagccgg
atgttcttgtgtccgaaattggtgggttcttggtctcactgacttcaaga
atgaagttgcggaccctcacggtgagtggtacagttcttaaagatgatgt
gtccagagtttgttccttctgatgttcggacgtgttcagagttacctcct
tctggtggattcgtggtctcgctggcttcaggagtgaagctgcagaccttt
gcggtgagtgttacagctcttaaggcggcatgtctggagtttgttcgtt
cctcccgtctggagttgttcattcctcctggtgggttcgtggtctcgctg
gcttcaggagtgaagctgcagacctctgcggtcggtgttaccagcagata
aatgctatgcggacccaaagagtgagcagcagcaagatttattgcaaaga
gcacaagaacaaagcttccacagcgtggaaggagaccagagcgggttgct
gctgctggctcaggcagcctgcattttttttttttttttttttttttttt
gagatggagtctccctctgtcacccaggctggaatgcagtggtgcaatct
gggctcactgcaagctccgcctcccgggttcacgccattctcctgcctca
acctccccagtagaggggattacaggcacccaccaccgcacccagctaat
attttgtctttttagtagagtcggggtttcactgtgttagccaggatggt
ctcgatctcttgacctcgtgatccacccctctaggcctcccaaattgctg
ggattacaggtgtgagccactggcacccagcggggcagcctgcttttatt
cccttatctgaccccacccacatcctgttgattggtccattttacagaga
gctaattggtccgttttgacagggtgctgattggtgcatttacaatccct
gagctagatatacacagagtgctgattggtgcatttacaatcctctagct
agacataaaaattctccaagtccccactacatttgctagacacagagcac
tgattggtgcgtttacaaacctttagctagacacagagtgctgattggtg
catttgcaaaccttgagctagacacagagcactgattggtgcatttacaa
tcctttagctagacacagaagttctccaagtgcccaccagattagctaga
tacagagtgctgattggtgcatccccaaaccccaagctagacacagagtg
ctgactggtgcatataaaatcctcaggctagacataaaagttttccaagt
ccccatctgactcaggagcccagctggcttcacctagtggatcctgcgca
gggctgtgccgggcgcctgcactcctctcagcccttgggcagtcgatggg
accgggcgctgaggagcagggggcggtgcccgtcggggaggctcaggcca
cgctggagctcacaggggttgggagggggctcgggcatggcgggctgcag
gtcctgagccttgccctgtgcagggcggctggggcccggtgagaattcaa
gcggggtgcaggcgggccggcagtgctgggggacccggcgcaccctctgc
agctgctggcccgggtgctaggcccctgactgcccggggccgggggtgcg
gggcccgctgagcccgcgcccacctggaactcgcgctggctggcgagcgc
tgcgcgcagccccagttcccacacccgcctctccctccacacttccccgc
aagcagagggagccggctctggcttcggccagcccagagaggggcccca
cagcgcagtggcgggctgaagggctcctccagcacggccagaatggacgc
caaggccgaggaggcgccgagagcgagcgagggctgctagcacgttgtca
cctcgcattctgaaccacagactctccaactctccggcgcttttcgccca
ctcggtccctcagaacacgaagggctctctcatcctgtcactaaaacgat
tagctgtccggagacacggaaaaagtcgcccctcttctttgcaggattcc
tcccttgaacttctccaaaccctcttagtgtgacgtgaccccacccctag
ctaacccaggctgcttccttaccagcttcccgccccctggggaggcggca
atgcaaagaccgtccgctgccagctctgccgctatctctgtggggtgaat
ctaacatggcggacaaagacagtaactagtcccgtttctccgcgttttcg
E2F4 Reg1 (ChIP)
ccaagaagattggctcttaccacttgtccctc***aaaacgaccaccccattg***
actggtggcgattgcgtcgacggagacggggcaaaagcaagctgaacccg
aaaaataacaaacactggggctgaggggtggaactacgagtgcgcagaca
tgggccagagcgcatttcccctgccccaggcaaattcggcgctcactgcg
tccccgcaggccactgaccttacaagactacttgccccagactcctggggc
tggatgggaattgtagtctccctaaagagttgtacgtatctttttaagg
cctagtttctgctttcaaaatacgaaaacataacactccagtccataact
gttgacaagtacaagcgcgcacaggtctccaatctatccactggatttcc
gtgagaattgtgcccgctctggtattggatgttcctctccataagactac
agtttctaaggaacactgtggcgaagacctttcattccgcaacgcatgct
ggaaataattatttccctccaccccccccaacaatccttattacttatattt
accgaaactggagacctccattagggcggaaagagtgggggattgggac
ctcttcttacgactgctttggacaataggtagcgattctgaccttcgtac
agcaattactgtgatgcaataagccgcaactggaagagtagaggctagagggc
aggcactttatggcaaactcaggtagaattcttcctcttccgtctcttt
ccttttacgtcatccgggggcagactgggtggccaatccagagccccg
agagacgcttggctctttctgtccctcccatcctctgattgtaccttgattt
cgta***ttctgagaggctgctgctta***gcggtagccccttg**gtttccgtgg**
E2F4 reg2 (ChIP)
caacggaaaagcgcgggaattaca**gat**aaattaaaactgcgactgcgcgg
cgtgagctcg***ctgagacttcctggacggg***ggacaggctgtggggtttctc
gtaagaatgatataaccaaaaggtatataatttggtaatgatgctaggtt

ggaagcaaccacagtaggaaaaagtagaaattatttaataacatagcgtt
cctataaaaccattcatcagaaaaatttataaaagagtttttagcacacagt
aaattatttccaaagttattttcctgaaagttttatgggacatctgcc
ttatacaggtattagaaacttactgcctttctctaatgcttctagtgtaa
aaacttgcagacttatgtaaagtagggctgtatcgccgtgcccccattgt
ctgttaatcttgtttttatatttttgattgtgtttccttttcttttttttt
ttttttttaagacagggtcctgctctgtcactgaggctggagtgcagtg
gcgtgatctcggctcactgtagcctctgtctcccagcctcttcctgcctt
agcctcccaaatagctgggactacaggcacacgctaccatgcccggccaa
tttttgtatttttttgtagagatgaggttttaccatgttgcccaggctggt
aactcctgagctcaggtgatctgcccacctcggcctcccaaagtgctggg
gttcacaggtgtgtgtttatttctatctaattatttacacaaacacaatg
tatttatatattgtgtatctcttctgctacaatgtaaattctatgagagt
agtaattttgtctgtctcaacactgtttttcctaagtttggtacatagta
ggcactcag**ATGCTT**aaaggaatgaatgaattgtgctttaattccacttt
TSS:TranscriptionalStartSite
actaaacccaaatctccctttggacattgttatctatgtgttttcaaaga
agtataatcataatttgacagaaatccttgagaggcagaactaagtgagg
gattgggcagggttcagatgttaagaacagtaagctcagcagggtgtgat
tgctcatgcctataaccctagcactctaggaggctgaggtgggatgattgctt
gaggccaggagtttgaaatcagcctgggcaacatagtgagaccccat
cactaccaacaaaataaataaataaatgtacatggtggcatatgcccatagt
cctagctacttgggaggctatagtgggaggatagcttgagtacagaagt
ctgaggctgcagtgagctatgattgtggcactgcatgctagcctgggca
atagagcaagaccctgtctctaaattaaacaaaaaaaaaaagtactctagttt
tctatgcaatgcattatatctgctgtggatttagggcagtattatatc
agataattttaggcatttggtaggcttaaatgaatgacaaaaagttacta
aatcactgccatcacacggtttatacagatgtcaatgatgtattgattat
agaggttttctactgttgctgcatcttatttttatttgtttacatgtcttt
tcttattttagtgtccttaaaaggttgataatcacttgctgagtgtg
tttctcaaacaatttaatttcagGAGCCTACAAGAAAGTAC
GAGATTTA First Exon
GTCAACTTGTTGAAGAGCTATTGAAAATCAT
TTGTGCTTTTCAGCTTGA
CACAGGTTTGGAGTgtaagtgttgaatatcccaagaat
gacactcaag

E2F4 Reg1 primers used in qRT-PCR of the ChIP experiments (Underlined in the DNA sequence above)

Using 1-based sequence positions

OLIGO	start	len	tm	gc%	any	3′	seq
LEFT PRIMER	1	21	61.06	47.62	4.00	0.00	ctaacatggcggacaaagaca
RIGHT PRIMER	100	18	59.19	50.00	3.00	0.00	caatggggtggtcgtttt

SEQUENCE SIZE: 100
INCLUDED REGION SIZE: 100

PRODUCT SIZE: 100, PAIR ANY COMPL: 4.00, PAIR 3′ COMPL: 3.00

```
1 ctaacatggcggacaaagacagtaactagtcccgtttctccgcgttttcgccaagaagat
  >>>>>>>>>>>>>>>>>>>>>
61 tggctcttaccacttgtccctcaaaacgaccaccccattg
                      <<<<<<<<<<<<<
```

E2F4 Reg2 primers used in qRT-PCR of the ChIP experiments (Underlined in the DNA sequence above)

OLIGO	start	len	tm	gc%	any	3′	seq
LEFT PRIMER	7	20	59.05	50.00	5.00	3.00	ttctgagaggctgctgctta
RIGHT PRIMER	129	19	60.80	63.16	5.00	3.00	cccgtccaggaagtctcag

SEQUENCE SIZE: 150
INCLUDED REGION SIZE: 150

PRODUCT SIZE: 123, PAIR ANY COMPL: 6.00, PAIR 3′ COMPL: 2.00

```
1 ttcgtattctgagaggctgctgcttagcggtagccccttggtttccgtggcaacggaaaa
        >>>>>>>>>>>>>>
61 gcgcgggaattacagataaattaaaactgcgactgcgcggcgtgagctcgctgagacttc
                                                 <<<<<<<<<<
121 ctggacgggggacaggctgtggggtttctc
      <<<<<<<<<
```

In red, E2F4 consensus sequences, E2FA (+) and E2FB (+) respectively, described in the paper of Bindra et al. 2005 Cancer Res. Figure 2A.

SUPPLEMENTAL INFO_1

The random amplified polymorphic DNA (RAPD) assay

The RAPD technique (Random Amplification of Polymorphic DNA) originally described by Williams et al., (1990) is used to assess also small changes in the genomic template during the proliferation of the tumor cells carrying mutant p53 proteins. Every nucleotide variation (insertions, deletions, DSBs, translocations, missense mutations) between different sets of template DNAs will result in the presence or absence of bands due to changes in the priming sites. The random amplified polymorphic DNA (RAPD) assay has been shown to detect genotoxin-induced DNA damage and mutations. The changes occurring in RAPD profiles include variation in band intensity as well as gain or loss of bands. It is a type of PCR reaction, but the segments of DNA that are amplified are random.

The scientist performing RAPD creates several arbitrary, short primers (8–12 nucleotides), then proceeds with the PCR using a large template of genomic DNA, the primers are able to differentiate between genetically distinct individuals or cell polpulations, although not necessarily in a reproducible way. No knowledge of the DNA sequence for the targeted genome is required, as the primers will bind somewhere in the sequence, but it is not certain exactly where. If two template genomic DNA sequences are different, the PCR products display different banding patterns.

In the field of cancer research, the RAPD assay and related techniques allow the simultaneous detection and cloning of genomic alterations, without previous knowledge of the altered region. The RAPD is very likely to detect genomic instability, because the cancerous cell will produce a clone of dividing daughter cells. the first studies using the RAPD assay for the detection of genomic instability in brain and lung cancer were performed by Dil-Afroze et al. [Dil-Afroze et al., Gene 1998] and Ong et al. [Ong et al., Carcinogenesis 1998], respectively. The detected alterations in brain tumor DNA included the loss of a normal band, appearance of a new band and amplification of a pre-existing band. More recently, the RAPD assay was used to assess the overall genomic instability in liver cancers in transgenic mice hepatocellular carcinoma and oral squamous cell carcinomas. In other studies, it has shown the ability of RAPD to detect DNA sequences representing genetic alterations in stilbene estrogen-induced cancer cells, skin cancers, colorectal tumors, head and neck squamous cell carcinoma, mouse hepatoma cells MH-22a, breast andWilm's human cancer tissues (Atienzar and Awadhesh, Mutation Research, 2006. Review).

However, the RAPD is a semi-quantitative method and the nature and amount of DNA effects can only be fully elucidated if changes occurring in RAPD profiles are specifically analysed (e.g. cloning, sequencing, probing).
